# Accelerated Catalyst Development via Kinetically Controlled Solid‐State Laser Synthesis and Automated Electrochemical Screening

**DOI:** 10.1002/smll.202511035

**Published:** 2025-12-26

**Authors:** Mattis Goßler, Huize Wang, Joanna Przybysz, Faisal Gabi Sameer Aldabain, A. Lucía Morales, Andreas Körner, Andreas Göpfert, Andreas Hutzler, Serhiy Cherevko, Marc Ledendecker

**Affiliations:** ^1^ Helmholtz Institute Erlangen‐Nürnberg For Renewable Energy Forschungszentrum Jülich GmbH Erlangen Germany; ^2^ Campus Straubing For Biotechnology and Sustainability, Sustainable Energy Materials Technical University of Munich Straubing Germany; ^3^ Department Chemical and Biological Engineering Friedrich‐Alexander‐Universität Erlangen‐Nürnberg Erlangen Germany

**Keywords:** accelerating catalyst development, automation, electrocatalysis, laser‐induced synthesis, nanoparticles

## Abstract

Wet chemical methods are widely employed for nanoparticle synthesis. While offering a high degree of tunability, they often require surfactants, high‐temperature processing, and large solvent volumes. Here, we present a rapid, ambient‐condition laser‐based synthesis method for supported metal nanoparticles, enabling precise control over particle size and distribution homogeneity, the critical parameters in catalytic performance. By tuning laser fluence through power and scanning speed, we systematically explore the crystallite size distribution of platinum, palladium, and iridium nanoparticles supported on carbon. To accommodate the potentially high‐throughput nature of laser synthesis, X‐ray diffraction (XRD) data are analyzed via automated Rietveld refinement, which provides fast, consistent, and quantitative insights into crystallite size and distribution, further validated by scanning transmission electron microscopy (STEM). Real‐time thermal imaging reveals material‐specific decomposition thresholds, correlating laser parameters with nanoparticle formation dynamics. Integration with an automated scanning flow cell inductively coupled plasma mass spectrometry (SFC‐ICP‐MS) platform demonstrates the method's compatibility with high‐throughput electrochemical screening, exemplified by stability testing of platinum catalysts for the oxygen reduction reaction (ORR). This approach offers a scalable and generalizable route for rapid catalyst discovery across diverse materials systems.

## Graphical abstract

A rapid laser‐based method produces metal nanoparticles on carbon under ambient conditions, eliminating the need for solvents and surfactants. By adjusting laser parameters, particle size and uniformity are precisely controlled and rapidly analyzed using X‐ray diffraction. The approach integrates automated characterization and electrochemical testing, enabling the potential for high‐throughput discovery and evaluation of efficient and stable catalysts.

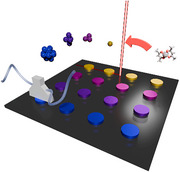

## 1 Introduction

The transition to sustainable energy systems hinges on the development of efficient and cost‐effective electrocatalysts for key technologies such as fuel cells, water electrolyzers, metal–air batteries, and electrochemical CO_2_ and nitrogen conversion [1, 2, 3]. Electrocatalysts lower activation energy barriers and modify reaction pathways, thereby enhancing charge‐transfer kinetics and reducing the energy input required for electrochemical energy conversion and storage systems. Central to these applications is the rational design of nanostructured catalysts, particularly noble metal nanoparticles supported on high‐surface‐area carbon materials. These supports enhance dispersion, prevent agglomeration, and facilitate electron transport, all of which are critical for catalytic performance [4]. However, achieving narrow and reproducible size distributions, especially in the sub‐5 nm regime, remains a significant challenge. While wet‐chemical synthesis methods offer tunability, they often require surfactants, high‐temperature processing, and large solvent volumes, and may suffer from limited scalability and reproducibility [5, 6]. Laser‐induced synthesis has emerged as a promising alternative, offering rapid, localized heating and tunable energy input under ambient conditions. Techniques such as laser ablation in liquids and laser‐induced direct writing have enabled the synthesis of diverse nanomaterials, including noble metals, alloys, and oxides [7, 8, 9, 10, 11, 12, 13]. A major advantage of laser‐based synthesis lies in its unique ability to create surface defects and unique topologies. In laser‐engineered materials, the extreme conditions of rapid heating to plasma/vapor states followed by ultrafast quenching generate high‐density defects (including stacking faults, grain boundaries, dislocations, and atomic steps) that remain kinetically trapped in the nanostructure [14, 15]. Yet, precise control over nanoparticle size and distribution remains difficult due to the complex interplay of laser parameters, precursor chemistry, and rapid thermal dynamics. While the above‐mentioned advantages were primarily observed in liquid‐state synthesis, we aim to develop our solid‐state synthesis to utilize similar, non‐equilibrium effects, with the complementary advantages of direct spatial patterning without post‐synthesis collection and rapid integration with characterization platforms. To fully exploit the temporal efficiency and flexibility of laser‐induced solid‐state synthesis, equally rapid and informative characterization methods are required. XRD, particularly when combined with Rietveld refinement, provides valuable insight into crystallite size distribution, complementing local imaging techniques such as STEM. Moreover, integrating synthesis with automated electrochemical evaluation platforms enables high‐throughput screening of catalyst performance, accelerating the discovery of optimized materials [16, 17, 18, 19].

In this work, we present a rapid and ambient‐condition laser‐induced solid‐state synthesis method for supported metal nanoparticles. By tuning laser fluence through power and scanning speed, we demonstrate precise control over crystallite size and distribution for platinum, palladium, and iridium nanoparticles on carbon supports. XRD, in combination with automated Rietveld refinement fitting, validated by high‐angle annular dark‐field scanning transmission electron microscopy (HAADF‐STEM), reveals material‐specific trends in particle formation, which are further interpreted using thermal imaging. Finally, we integrate this synthesis approach with an automated scanning flow cell ICP‐MS platform to assess the electrochemical stability of platinum catalysts for the oxygen reduction reaction (ORR). This methodology is readily adaptable to multi‐component systems and diverse supports, offering a scalable route toward high‐throughput catalyst discovery.

## 2 Results and Discussion

### 2.1 Laser‐Induced Synthesis

To demonstrate the validity of the laser‐based synthesis approach, platinum on carbon (Pt/C) was selected as a model system. Platinum serves as the benchmark catalyst for electrochemical reactions, with Vulcan XC‐72R chosen as the widely used support material [20, 21, 22, 23]. Platinum(II) acetylacetonate (Pt(acac)_2_) was selected as the metal precursor due to its clean thermal decomposition, which reduces the metal while leaving behind only intact ligands or organic fragments, an advantage over halide‐based alternatives [22, 24, 25, 26]. Furthermore, we observed a substantially lower degree of particle size control when using K_2_PtCl_4_ as a precursor, as shown in Figure S1.

**FIGURE 1:**
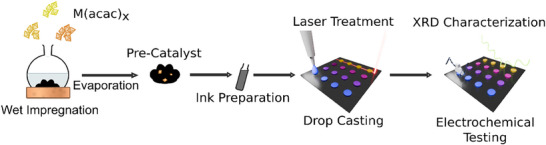
Schematic representation of the synthesis and characterization workflow, including the impregnation process, deposition on the substrate, and laser‐induced pyrolysis of the precursor.

The carbon black (CB) was impregnated with the precursor to yield a pre‐catalyst containing 10 wt% platinum. After impregnation, the catalyst was drop‐cast to produce a thin film, as depicted in Figure 1, which is subsequently treated with the CO2 laser. CO2 laser sources emit coherent light in the far infrared (IR) regime with a wavelength of 10.6 µm. Figure S2 compares the optical absorption of the carbon support and the metal–organic precursor. The CB exhibits strong absorption at the laser wavelength, whereas the precursor absorbs only weakly. As a result, the dominant reaction pathway is photothermal: laser irradiation induces lattice vibrations in the CB, which transfer thermal energy to the adsorbed precursor molecules, triggering their decomposition. To systematically study and control the laser–material interaction, we introduce the concept of fluence, a key parameter in laser processing. Fluence quantifies the energy delivered per unit length or area and directly influences the extent of thermal decomposition. It is defined as:

(1) $$F_{L}=\frac{P}{v}\;F_{A}=\frac{P}{v\cdot d}\;\;$$

with *P* being the laser power, *v* being the scanning speed, and *d* being the laser spot diameter. Table S5 summarizes the lineal fluence *F_L_* (given in J m^−^) and areal fluence *F_A_* (given in J m^−2^) values, resulting from the laser powers and speeds used. By adjusting these parameters, the energy input can be precisely tuned to optimize catalyst formation.

### 2.2 Crystallite Size Distribution From XRD Analysis

To see at which minimal fluence the pyrolysis of precursor begins to set in, the fluence was gradually increased by selecting a low laser power of 0.14 W and decreasing the scanning speed from 1.30 m s^−1^ to 0.11 m s^−1^. The flexibility and swiftness of the laser‐induced synthesis allow for a fast screening of process parameters, which must be combined with an equally fast characterization method to unleash its full potential.

**FIGURE 2:**
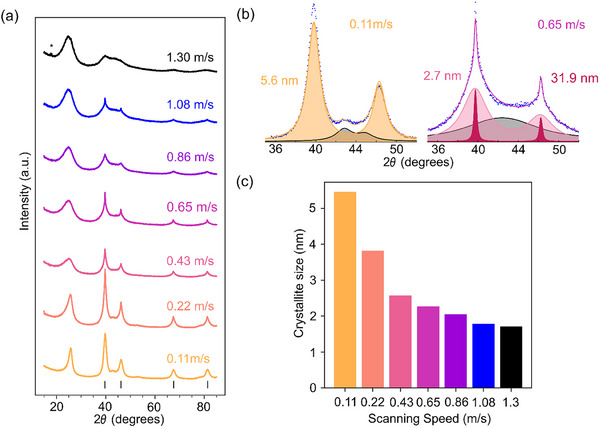
Effect of scanning velocity on crystallite size distribution in synthesized catalysts. (a) XRD patterns of catalysts synthesized at varying scanning speeds, using 0.14 W power. (b) Representative peak fittings illustrate unimodal (0.11 m s^−1^) and bimodal (0.65 m s^−1^) crystallite size distributions, highlighting the difference between narrow and broadened size populations. The contribution of the carbon support is accounted for, indicated by the gray curve between the metal reflexes (≈43°). (c) Extracted mean crystallite sizes of the major fraction as a function of scanning velocity, demonstrating a systematic increase in particle size with increased fluences.

XRD was employed as a rapid and informative tool to assess crystallite size and distribution, and the emergence and sharpening of characteristic Pt features are shown in Figure 2a. The scanning speed gradually decreased from 1.30 m s^−1^ to 0.11 m s^−1^, increasing the fluence from 0.93 kJ m^−2^ to 11.11 kJ m^−2^. As the energy input increases, the characteristic (1,1,1), (2,0,0), (2,2,0), and (3,1,1) reflexes of the face‐centered cubic (FCC) platinum crystal structure emerge and gradually sharpen. Rietveld refinement revealed that a single‐component fit was insufficient at intermediate fluences to describe the diffraction profiles.

Instead, a two‐component model, representing fine and coarse crystallite populations, yielded significantly improved fits (Figure 2b). This suggests a broad or bimodal size distribution, common in non‐equilibrium synthesis processes. Commonly, reflexes in XRD patterns are assumed to be of Gaussian shape, Lorentzian shape, combinations thereof, or similar functions [27]. These are used to describe populations with narrow crystallite size distributions, and a Rietveld refinement with only one component will not reach sufficient accuracy when the distribution is too wide or even multimodal [28, 29]. Such distributions are indicated by a so‐called ‘super‐Lorentzian’ line shape, exhibiting a broader base and sharper top as shown in Figure 2b. They can only be sufficiently fitted with a peak model including a component for the finer and one for the coarser fraction of metal crystallites. As the intensity in X‐ray diffraction scales cubically with respect to the number of scatterers in the coherently scattering domains, larger crystallites will dominate the intensity in such multimodal distributions, and crystallite size extraction utilizing a simple Scherrer equation will easily disregard the contribution of small crystallites, even if their number density is in the majority [30]. As small crystallites contribute the most to the catalytically active surface area, it is essential to properly account for them when correlating characterization results with catalytic testing.

Care must be taken in interpreting these results, as a fit with two components yields two discrete crystallite size populations with two mean crystallite sizes. This does not mean that the material necessarily exhibits a real bimodal size distribution, but it is instead an indicator of the broadness of the size distribution. For artificially created bimodal crystallite size mixtures, it was shown that fitting with two components yielded reliable results for the crystallite sizes of the fine and coarse fractions and their mass percentages in the mixture. Importantly, the size of the coarse fraction was systematically overestimated. The analysis is less reliable for crystallite sizes that lie closer together, where a single‐component fit is more appropriate [29].

In this analysis, the decision whether one or two components were used for the Rietveld refinement was made based on the shape of the error curve, where a symmetrical ‘W’ shape indicates a mismatch of the profile shape. In these cases, the broadness of the base of the reflex is underestimated, while the broadness of the top is overestimated, as shown in Figure S3. If this shape mismatch couldn't be resolved by just considering peak and strain broadening, a two‐component fit is justified.

### 2.3 Comparison of XRD and STEM Results

**FIGURE 3:**
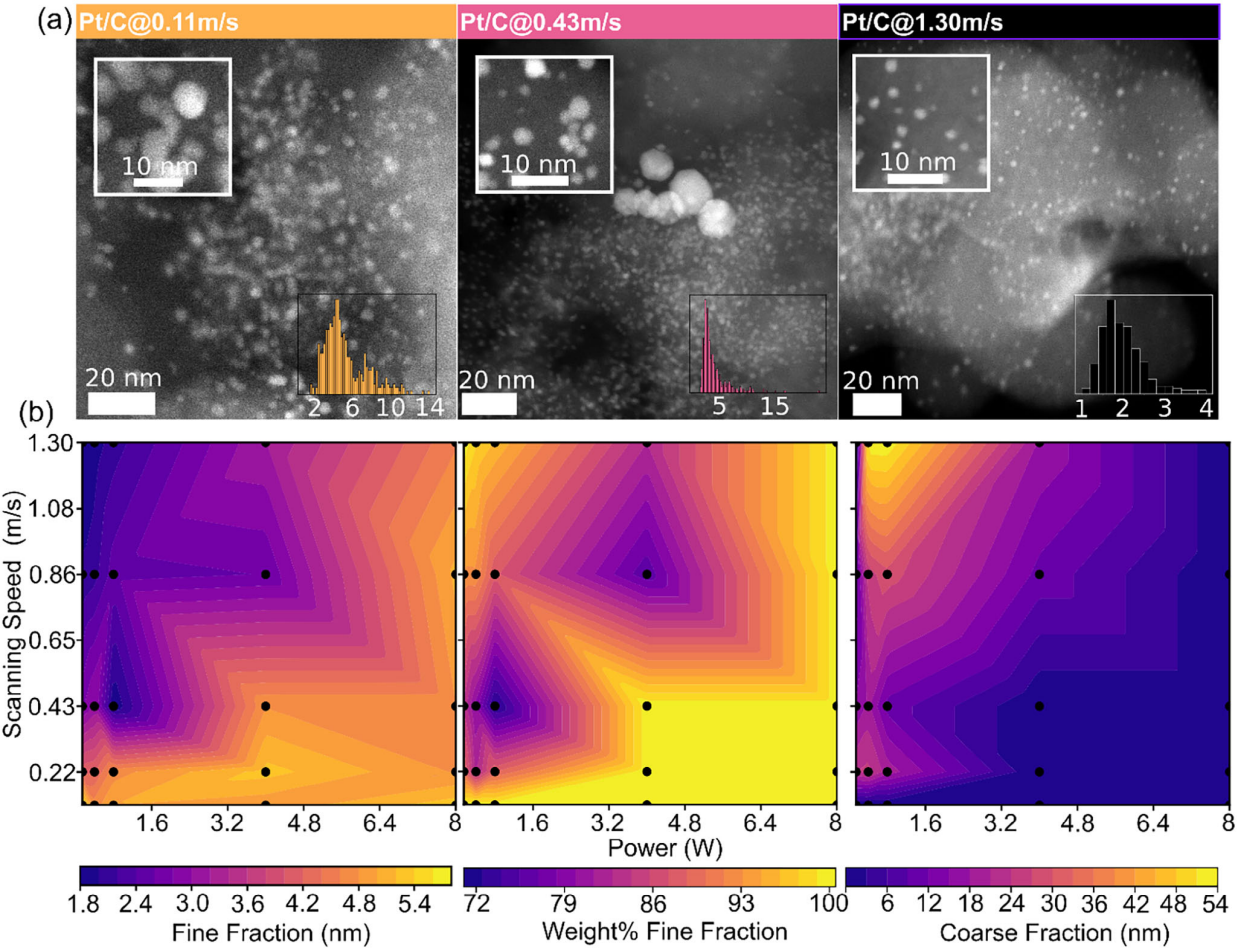
Crystallite sizes and homogeneity of platinum particles at various laser parameters, analyzed by XRD and STEM. (a) HAADF‐STEM images of catalysts produced at the indicated scanning speeds and 0.14 W power. The insets in the lower right corners show particle size distributions, gathered from multiple images per sample, and around 200–300 particles per sample. The insets in the upper left corner show close‐ups of representable particles from each population. (b) From left to right: crystallite sizes of the fine fraction, weight percentage of the fine fraction, and crystallite size of the coarse fraction, obtained from Rietveld refinements. The weight percentage of the fine fraction can be interpreted as a metric for the homogeneity of the distribution and whether the fine or the coarse fraction is more representative of the material. Between measured data points, linear interpolation has been used to produce the contour plots to better visualize trends.

Using the above‐described analysis, the crystallite sizes of the fine fraction, which was the major component for each of these cases, were extracted and shown in Figure 2c. A systematic correlation can be observed, where higher fluences lead to larger mean crystallite sizes, and a secondary trend can be observed, where the crystallite size distribution broadens when transitioning from very low to intermediate fluences, and then sharpens again to a more homogenous size distribution at high fluences, fitted with only one component. This same trend was verified by HAADF‐STEM, which is shown in Figure 3a for three cases. For all the TEM analyses, 200–300 particles were evaluated, their Feret diameter was extracted, and used to calculate the mean Sauter diameter. The latter is essential for catalysis, as it directly relates to the active surface area available for reactions. As the obtained sizes are compared to the fine fraction of the XRD analysis, outliers are filtered before calculating the mean for samples with broader size distributions using a median absolute deviation approach.

In all HAADF‐STEM images, well‐dispersed particles on the carbon support with different size distributions could be observed. For a low fluence at a scanning speed of 1.30 m s^−1^, particles with a narrow mean size of 2.1 nm are observed, while the XRD analysis yielded an average crystallite size of 1.6 nm. At an intermediate fluence at a speed of 0.43 m s^−1^, a mean particle size of 3.9 nm is extracted for the major phase from the STEM images. At the same time, larger particles can be observed, verifying the broad size distribution predicted from XRD analysis. The latter yielded a crystallite size for the fine component of 3.2 nm. At the highest fluence resulting from a scanning speed of 0.11 m s^−1^, the size heterogeneity decreases again, no particle size outliers were observed, and the distribution centers around 6.0 nm, again compared to the XRD fitting result of 5.6 nm. The crystallite sizes obtained from the XRD analysis are systematically smaller by 6% to 25% (Table S1) compared to the particle sizes from the TEM analysis. This is expected, as STEM visualizes individual particles, which can be polycrystalline, whereas XRD measures the coherent scattering domains [29].

While XRD provides statistically averaged information, STEM is inherently local. Recovering particle size distributions from XRD using STEM is impractical for heterogeneous samples, as it would require counting a prohibitively large number of particles. Despite this limitation, the described XRD analysis offers a rapid and effective means to navigate the broad parameter space of the laser synthesis process. It captures trends in crystallite size, correlating with particle size, and provides insights into distribution homogeneity.

### 2.4 Crystallite Size and Homogeneity of Platinum Particles in the 2D Fluence Space

After it has been established that the crystallite size can be tuned by varying the fluence through the scanning speed, the natural extension is to explore varying laser powers. To explore the whole synthesis space, a 5 × 5 grid of laser powers and scanning speeds was investigated. As the scanning speeds used before have proven to produce a range of crystallite sizes, five of these (1.30m s^−1^, 0.86m s^−1^, 0.43m s^−1^, 0.22m s^−1^, 0.11m s^−1^) have been chosen and were appended by five laser powers (0.14 W, 0.4 W, 0.8 W, 4 W, 8 W). While the same laser fluences can be produced by either changing the laser power or scanning speed, as seen in Equation (1), it is not to be assumed that the effect on the material is invariant when choosing either of these options. While lower power and lower speed will lead to a slower and more sequential heating effect with more heat dissipation, the same energy input at higher speeds and powers could lead to a thermal shock with faster dynamics, which can significantly influence decomposition and diffusion processes of the material. This, in turn, has been shown to change resulting particle sizes non‐linearly [31, 32]. Furthermore, varying the scanning speed while maintaining a constant number of incident laser pulses affects the interaction time with the material. Slower speeds result in longer pulse durations and extended energy transfer, further influencing the thermal profile and reaction kinetics.

To efficiently evaluate the increasing number of XRD data and to rationalize the decision for a one‐ or two‐component fit, an automated approach for Rietveld refinement has been developed. Within this framework, every pattern is automatically fitted with both models, and the quality of the fits is judged by evaluating their χ^2^‐values. If a two‐component fit improves the χ^2^‐value by at least 10%, it is assumed to outweigh the additional complexity of the model and is accepted by the algorithm. This accurately reproduces the manual decision based on the aforementioned peak‐shape misfit, thus significantly decreasing the time needed for data evaluation.

The results of the XRD analysis conducted on all 25 samples, synthesized with the above‐mentioned laser parameters, are summarized in Figure 3b. The three contour plots show the obtained crystallite size of the fine fraction, the fine fraction's weight percentage, and the coarse fraction's crystallite size, from left to right, respectively. A systematic crystallite growth of the fine fraction from lower to higher fluences is observed. In all cases, the fine fraction is in the majority, independent of laser parameters, with the lowest weight content and thus greatest heterogeneity being 71% at intermediate laser fluence (0.8 W, 0.43 m s^−1^, 15.43 kJ m^−2^). This suggests that very homogeneous particle size distributions are achievable, and particle sizes can be tuned by carefully choosing laser parameters. Looking at the coarse fraction's crystallite sizes, one can identify that the low fluence region has some instability, by either producing a homogenous population of small crystallites (1.6 nm at the low fluence end) or a mixture of these and a minimal phase content of larger crystallites (≈ 2%). As this fluence region constitutes the transition from solely precursor composition to the early stages of particle growth, care must be taken to obtain a homogeneous sample. However, very low mass contents of larger particles are not expected to significantly alter the overall catalytic performance. Remarkably, while intermediate fluences produce broader size populations with larger heterogeneity, the usage of high fluences narrows the distribution to a homogenous mean crystallite size of around 5.6 nm. The qualitative behavior shown in Figure 2a, where the power was kept constant and only the speed was changed, is thus retained but generalized to the speed‐power parameter space.

### 2.5 Generalization of the Synthesis Approach to Other PGM Particles

**FIGURE 4:**
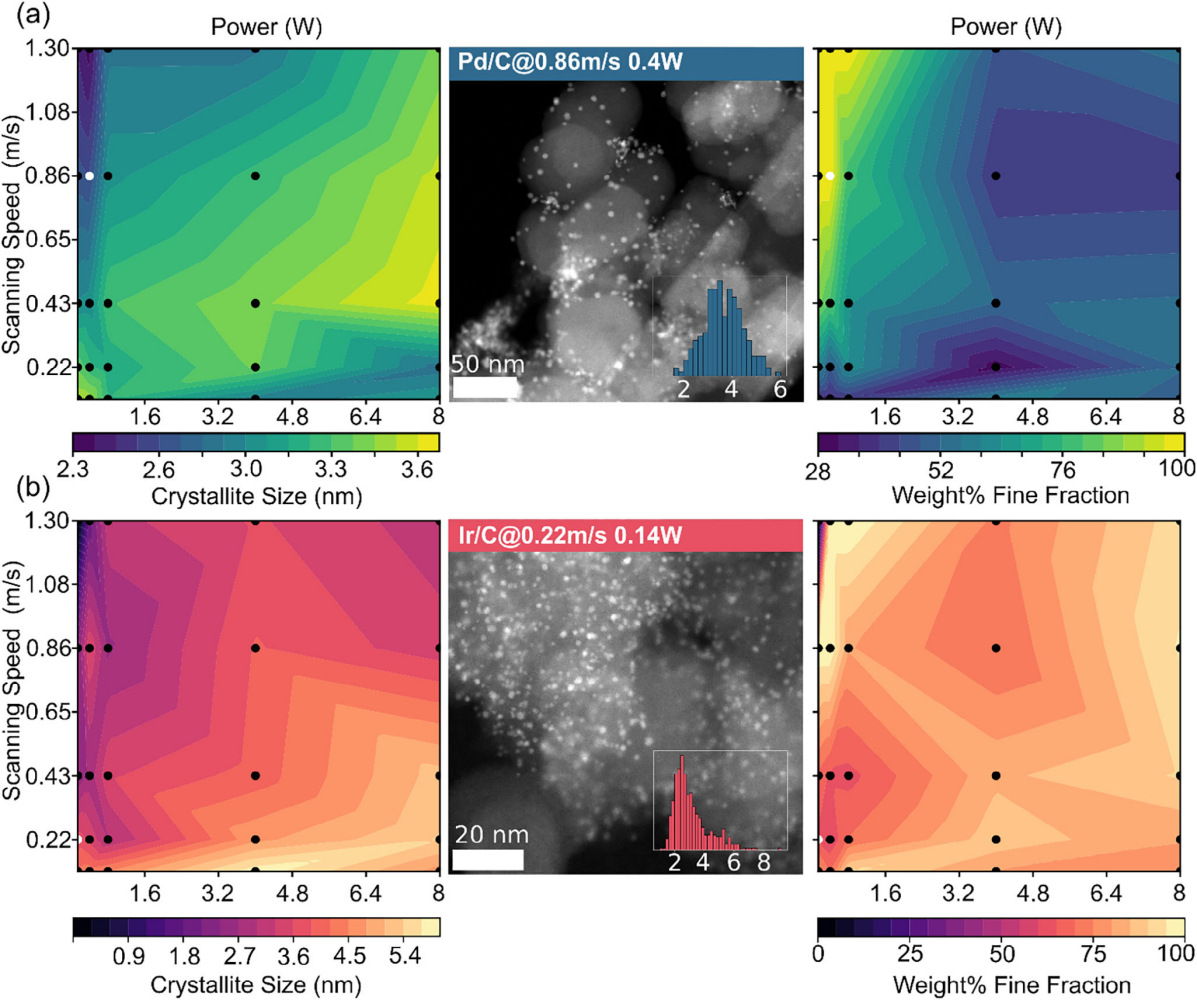
Palladium/Carbon (a) and Iridium/Carbon (b) catalysts, produced from various laser parameters. The crystallite size of the fine fraction is shown in the left column. The middle column shows HAADF‐STEM images of Pd/C and Ir/C catalysts, obtained at selected synthesis conditions (indicated by white spots in the contour plots) to showcase well‐dispersed particles with differing size distributions. The right column shows the weight fraction of fine crystallites in the catalysts, a metric for the homogeneity of the materials.

To demonstrate generality, the synthesis was extended to palladium and iridium using Pd(acac)_2_ and Ir(acac)_3_ precursors. As shown in Figure 4, crystallite sizes, and weight fractions of the fine fraction were mapped across laser parameters. Both systems exhibited similar trends in crystallite size evolution, though with metal‐specific differences. For Pd/C, crystallite sizes ranged from ca. 2.4 to 3.6 nm, but heterogeneity increased sharply at intermediate fluences, with the fine fraction dropping to ≈ 30 wt%. At high fluence, similar to Pt, the size distribution narrows, and mass percentages of 30% to 60% can be reached. Ir/C showed a broader size range (ca. 2.0–5.9 nm) and achieved high homogeneity (≈ 96%) at high fluences. While particles formed under all conditions for Pd, no XRD reflexes were observed for Ir at low fluences, likely due to insufficient decomposition temperature or subcritical particle sizes.

### 2.6 Real‐Time Thermal Imaging of the Laser Process and Decomposition Chemistry of Acetylacetonates

Thermal imaging was employed to better understand these metal‐specific particle formation differences and correlate synthesis conditions with thermal decomposition behavior. This technique provided real‐time temperature profiles during laser irradiation, offering insight into whether the temperatures achieved were sufficient to trigger precursor decomposition. In air or inert atmospheres, Pt(acac)_2_ begins to decompose around 200 °C, with thermogravimetric analysis (TGA) showing rapid mass loss between 200–320 °C due to ligand breakdown. The process is mainly atmosphere‐independent, indicating that ligand removal, rather than oxidation, is the dominant mechanism. Within this temperature window, decomposition can occur in solid, liquid, or vapor phases [33]. For Pd(acac)_2_, non‐isothermal studies have shown decomposition occurs between 135–300 °C, with higher heating rates shifting the onset to higher temperatures [34]. A similar heating‐rate‐dependent decomposition behavior has been reported for Pt(acac)_2_. [26] In contrast, Ir(acac)_3_ exhibits a higher thermal stability, with decomposition initiating between 200–300°C and completing at even higher temperatures, depending on the conditions [35, 36, 37]. Within this temperature range, all three precursors may undergo phase transitions, including vaporization, which can influence decomposition pathways [38]. Given the rapid energy input of the laser process, orders of magnitude faster than conventional heating, melting, vaporization, and decomposition are expected to occur concurrently.

To understand the implications for nanoparticle growth, it is useful to consider the Hüttig (*T_H_*) and Tamman (*T_T_*) temperatures, which approximate the onset of surface and bulk atomic mobility, respectively. These thresholds mark the transition to regimes where sintering, agglomeration, and Ostwald ripening become significant. These effects are further amplified for nanoparticles due to size‐dependent melting point depression [39]. The relevant temperatures are *T_H,Pt_* = 339°C*, T_T,Pt_* = 747°C, *T_H,Pd_* = 275°C, *T_T,Pd_* = 641°C, *T_H,Ir_* = 543°C, and *T_T,Ir_* = 1087°C, as calculated from the relevant melting temperatures taken from literature [40].

**FIGURE 5:**
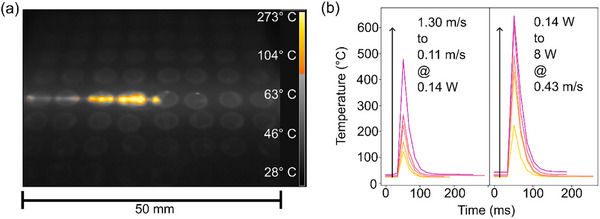
Estimates of the temperatures evolving in the thin films while undergoing laser treatment were obtained from thermal imaging. (a) Snapshot of the laser beam passing over a row of catalyst spots, drop‐cast onto a glassy carbon plate. The color bar indicates the temperatures evolving during laser treatment. (b) Temporal temperature evolution in the middle of a catalyst spot at different laser parameters. The temperature sharply increases for a frame (17 ms) and then decays exponentially.

To relate the thermal decomposition behavior of the precursors to the laser synthesis process, infrared thermal imaging was employed to estimate the temperatures generated within the catalyst films during irradiation. As shown in Figure 5a, laser treatment of a row of catalyst spots on a glassy carbon substrate resulted in visible light emission, indicative of localized heating. Peak temperatures were extracted for each spot and averaged to determine mean values across the array. Figure 5b presents the temporal evolution of temperature at selected laser parameters, chosen to span the whole fluence space by varying either scanning speed (0.11 m s^−1^ – 1,30 m s^−1^) at constant power (0.144 W), or laser power (0.144 W – 8 W) at constant speed (0.43 m s^−1^). Under these conditions, mean temperatures ranged from 129 °C to 481 °C (speed variation) and 221 °C to 666 °C (power variation), with corresponding peak temperatures reaching up to 704 °C, the upper detection limit of the thermal camera. As such, temperature values at higher fluences are likely underestimated. To overcome this limitation and extract more precise correlations between laser parameters and material properties, more advanced methods for temperature measurements could be applied in future studies. Luong et al. used a self‐developed device to extract accurate temperature values with high temporal resolution by fitting measured blackbody radiation during the flash Joule heating synthesis of graphene [41]. All values are summarized in Table S2.

The heating and cooling dynamics were extracted from the temperature‐time profiles and are shown in Figure 5b. The fits are displayed in Figure S16. All heating events exhibited a rapid, near‐instantaneous rise, limited by the 60 Hz frame rate of the camera, followed by exponential decay. Heating rates increased from 5.9 × 10^3^ °C s^−1^ (0.14 W, 1.30 m s^−1^) to 3.55 × 10⁴ °C s^−1^ (8 W, 0.43 m s^−1^), while cooling halftimes varied modestly from 10.6 ms to 15.3 ms across the tested conditions. It is important to note that each point in the film experiences multiple heating events due to the rastering pattern of the laser. While the highest temperatures occur when the beam directly irradiates a spot, adjacent passes contribute additional thermal input, resulting in an averaged thermal exposure across the film.

By combining literature‐reported decomposition thresholds with thermal imaging data, we can infer the mechanisms governing precursor decomposition and nanoparticle growth. At low fluences, laser‐induced mean temperatures below 200 °C are sufficient to initiate partial pyrolysis of Pt(acac)_2_ and Pd(acac)_2_, leading to the formation of small, immobile clusters on the carbon support. In contrast, Ir(acac)_3_ requires higher temperatures for decomposition, explaining the absence of detectable Ir particles at low fluences.

With increasing fluences, particle growth mechanisms are enabled by increasing reaction temperature, the first of which are coagulation and sintering of particles. When mean temperatures approach T_H_, particles become mobile, coagulate, and sintering begins to occur, mediated by mobile surface atoms. As the energy increases and approaches the T_T_ of the metals, atom mobility within the particles increases and allows for Ostwald ripening‐like mechanisms [10]. In this stage, atoms of smaller particles detach and redeposit on larger particles, driven by decreasing surface energy. While this process ideally leads to size homogenization, the rapid heating and cooling inherent to laser processing likely interrupt ripening prematurely, resulting in broader size distributions.

Metal‐specific differences in growth behavior were observed. Platinum tends to form more uniform particles at high fluences, suggesting it reaches a quasi‐equilibrium growth regime. Palladium, with its lower melting point and higher vapor pressure [40], exhibits greater size instability, likely due to early precursor decomposition and enhanced atomic mobility. Iridium shows intermediate behavior, achieving high homogeneity only at elevated fluences.

Jackson et al. studied the non‐isothermal decomposition of Pt(acac)_2_ at modest heating rates for the generation of nanoparticles in a closed reactor setup. They postulated that the pressure generated by decomposition products of the organic ligands may inhibit particle growth [33]. In the case of the current study, an open reactor was used, but the high local pressures generated at laser incidence could lead to a similar mechanism [42]. The local pressure might also depend on whether the ligands detach from the metal atom intact or decompose further, which is dependent on the nature of the complex and could also explain differences between the metals [26, 43].

### 2.7 Experimental Case Study With Automated Electrochemical Testing

**FIGURE 6:**
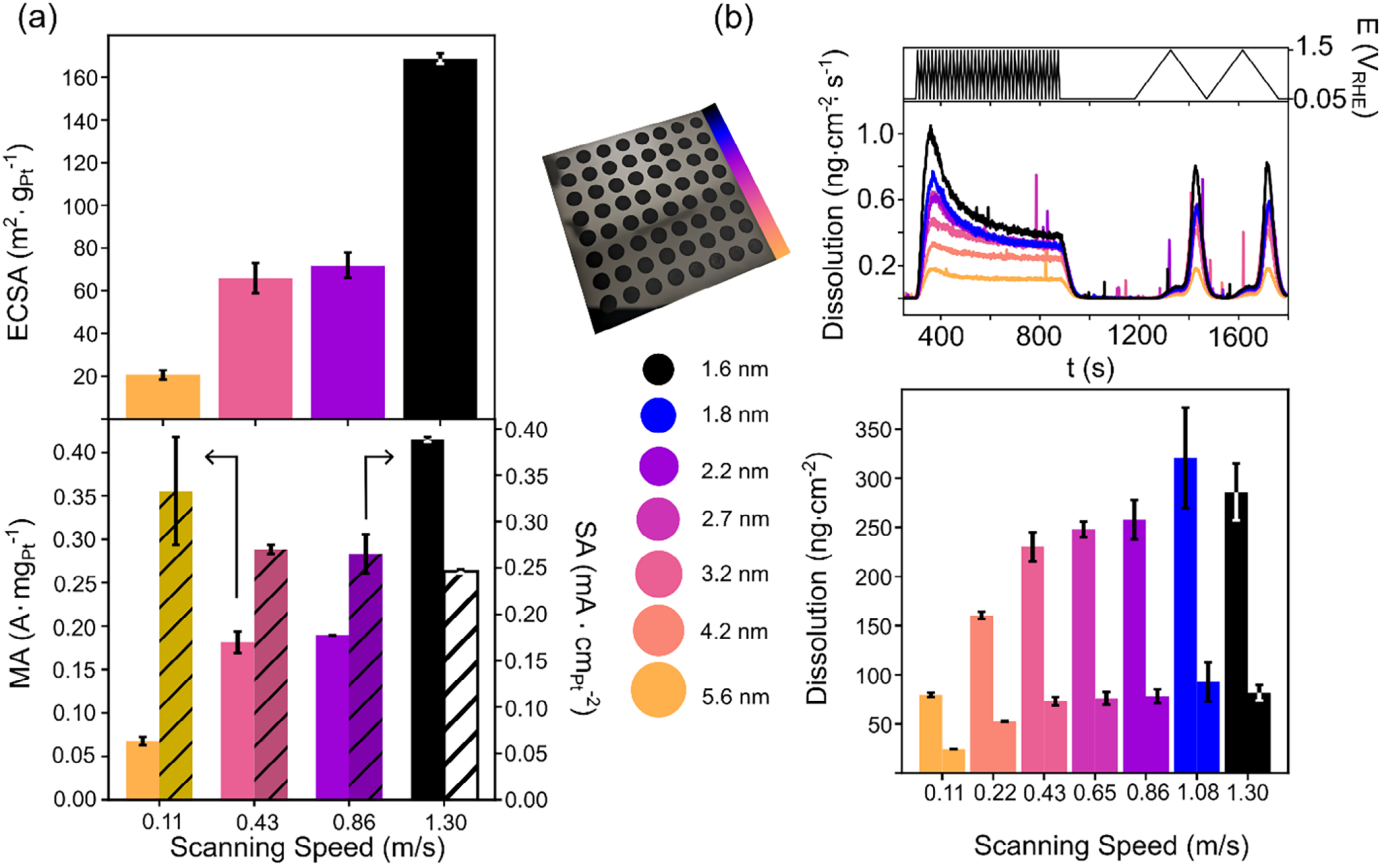
Electrochemical performance and stability assessment of laser‐synthesized Pt/C catalysts with varying scanning speeds. a) Top: ECSA derived from hydrogen underpotential deposition (H_upd_) regions in RDE measurements. Bottom: Corresponding MA and SA extracted from ORR polarization curves. b) Top: time‐resolved platinum dissolution profiles recorded via automated SFC–ICP–MS during 40 fast CVs (200 mV·s^−1^) and two slow CVs (10 mV·s^−1^) between 0.05 and 1.5 V_RHE_. Bottom: integrated platinum dissolution, separated into contributions from fast (left bars) and slow (right bars) CVs. The central panel displays a photograph of the GC plate (50×50 mm) with drop‐cast and laser‐treated catalyst spots, annotated with a color legend linking scanning speed to particle size, as defined in Figure 2.

To evaluate the catalytic relevance of laser‐synthesized Pt/C nanoparticles, we investigated their electrochemical performance and stability using a combination of rotating disk electrode (RDE) measurements and an automated scanning flow cell, coupled with inductively coupled plasma mass spectrometry (SFC–ICP‐MS). This approach enabled high‐throughput, time‐resolved analysis of platinum dissolution under dynamic potential cycling [17, 18, 19]. Catalyst films were prepared by drop‐casting a grid of pre‐catalyst spots onto a glassy carbon electrode, each synthesized under varying laser fluences to yield crystallite sizes ranging from 1.6 to 5.6 nm. A representative image of the GC electrode is shown in Figure 6 (middle column). Electrochemical characterization revealed that smaller particles exhibited higher electrochemically active surface area (ECSA) and mass activity (MA), consistent with increased surface‐to‐volume ratios (Figure 6a), a trend integrating well with a commercial benchmark catalyst (Figure S18). However, these benefits came at the cost of reduced stability: platinum dissolution increased markedly by a factor of more than three for 1.6 nm crystallites compared to 5.6 nm crystallites (Figure 6b). This trend aligns with literature and reflects the higher oxophilicity and surface energy of smaller particles, which promote oxidation and subsequent dissolution [44, 45, 46, 47]. Additionally, mass transport effects concurrently have a large effect on dissolution rates, where higher Platinum loadings, facilitated in this work by higher fluences, induce further stabilization, enhancing the demonstrated dissolution trend [48]. Specific activity (SA) showed a modest increase with particle size, indicating improved ORR kinetics due to reduced oxygen binding strength. Notably, intermediate laser fluences produced catalysts with a favorable balance of activity and stability, despite broader size distributions. These findings suggest that while larger crystallites contribute minimally to ECSA, they may facilitate Ostwald ripening, potentially stabilizing the catalyst at the expense of mass utilization [49]. Overall, this study highlights the trade‐off between activity and durability in nanoparticle catalysts and demonstrates the utility of laser synthesis for tuning this balance. The integration of automated electrochemical testing with time‐resolved dissolution analysis provides a powerful framework for rational catalyst design.

## 3 Conclusions

We have demonstrated a rapid and generalizable laser‐induced synthesis method for producing supported metal nanoparticles under ambient conditions. By leveraging tunable laser parameters, we achieved precise control over nanoparticle size and uniform distribution, enabling systematic exploration of structure–property relationships relevant to electrocatalysis. XRD‐based microstructural analysis, complemented by STEM, revealed that crystallite size and homogeneity can be finely tuned across a broad parameter space. The synthesis approach was successfully extended to palladium and iridium, highlighting its versatility. Thermal imaging provided insights into the photothermal decomposition mechanisms of metal–organic precursors, correlating laser fluence with reaction temperature and particle growth dynamics. Integration with an automated SFC–ICP–MS platform enabled high‐throughput electrochemical evaluation of catalyst stability. A clear size–stability relationship was demonstrated, with larger crystallites exhibiting significantly reduced platinum dissolution. RDE measurements further confirmed the size‐dependent trends in activity and utilization. Together, this study presents a scalable workflow for the synthesis, characterization, and evaluation of supported metal catalysts that shows great promise for automation. The methodology promises to be readily adaptable to multicomponent systems and diverse supports, resulting in a powerful tool for accelerating catalyst discovery and rapid testing towards optimization of materials in energy conversion technologies.

## 4 Experimental Section

### 4.1 Chemicals and Materials

Platinum (II)‐acetylacetonate (97%, Sigma‐Aldrich), Palladium (II)‐acetylacetonate (99%, Umicore), and Iridium (III)‐acetylacetonate (97%, Sigma‐Aldrich) were used as metal precursors. Vulcan XC 72R carbon black (Fuel Cell Store) served as the support material. Solvents included propan‐2‐ol (≥99.8%, Fisher Chemical), ethanol (≥99,8%, Thermo Fisher), acetone (≥99.8%, Fisher Chemical), and ultrapure water (18.2 MΩ·cm, Milli‐Q, Merck). All chemicals were used as received without further purification.

### 4.2 Impregnation to Obtain The Pre‐Catalyst

To ensure an even coating of carbon black (CB) with the precursor, we employed a wet impregnation technique. Metal salts were dissolved in acetone, mixed with the CB in a round‐bottom flask, and stirred overnight. The amount of metal precursor was calculated to obtain a catalyst with 10 wt% final metal loading (excluding the organic ligands). The solvent was then removed via rotary evaporation (Hei‐VAP Core, Heidolph), and the pre‐catalyst was obtained as a dry powder.

### 4.3 Film Deposition and Laser Treatment:

To obtain reproducible pre‐catalyst thin‐films with a thickness of 2 µm (monitored by laser microscopy, VK‐X1000, Keyence), a dispersion of 3 mg/ml pre‐catalyst was prepared by sonicating a dispersion of the powder in a solvent mixture of 100 µL ethanol, 100 µL isopropanol, and 800 µL water for 30 min. After sonication, for bulk production of catalyst, the dispersion was pipetted on a titanium sheet and left to dry on a hot plate at 60°C in air. After drying, the thin film was subjected to laser treatment. For the SFC‐ICP‐MS measurements, the dispersion was directly drop‐cast on glassy carbon (GC) plates (50 mm x 50 mm x 4 mm, Type 2, Alpha Aesar), which were polished for 5 min using a polishing machine (LaboForce‐100, Strues). The GC plates were then rinsed with ultrapure water. An 8×8 array of 5 µL spots was then drop‐cast onto the GC plates using an automated liquid handler (epMotion 5073, Eppendorf).

The laser treatments were performed using a CO2 laser (Speedy 400 Flexx, Trotec). It uses an RF tube with a wavelength of 10.6 µm, a maximal output power of 80 W, and a gantry system that can move over the sample with a maximal speed of 4.32 m s^−1^. The laser's spot size was 120 µm in focus. All samples were placed in the focal plane of the laser and treated with different laser powers and scanning velocities, denoted in percentages of the maximal values. The power varied from 0.18% to 10% (0.14 W to 8 W), and the scanning speed varied from 2.5% to 30% (0.11 m s^−1^ to 1.30 m s^−1^). The software adjusted the frequency of the laser pulses so that a constant pulse density of 1000 ppi (pulses per inch) was kept. For further characterization, the catalyst powder was scratched off the plate and washed by mixing the powder with acetone, sonicating it, and finally centrifuging it, after which the powder was dried again. The GC samples for SFC‐ICP‐MS measurements were treated similarly, with one row of spots treated with a single set of laser parameters to obtain duplicates for further measurements. The whole GC plate was washed by immersion in acetone for five minutes and subsequently dried.

### 4.4 X‐ray Diffraction

The X‐ray diffraction (XRD) data were recorded using a Rigaku SmartLab SE diffractometer equipped with a Cu Kα X‐ray source with a wavelength of 1.54 Å. Measurements were recorded in Bragg‐Brentano geometry over a 2Θ range from 30° to 80° with a step size of 0.05° and a measurement speed of 1° per minute. All samples were measured on low‐background silicon holders. For evaluation of the XRD data, Rietveld refinements have been conducted at first manually using Profex [27], which utilizes the BGMN kernel. BGMN's profile model deconvolutes the measured profile into contributions from the wavelength distribution, the instrumental function, and the sample function, from which microstructural parameters can be extracted. The instrumental function was simulated via raytracing using the specific goniometer parameters of the diffractometer in‐house, using the GEOMET package, which comes with BGMN. After accounting for the wavelength profile of the copper radiation and instrumental contributions, the sample function models the diffraction reflexes using a Lorentzian component for angle‐independent peak broadening due to crystallite size effects and a squared Lorentzian function for angle‐dependent peak broadening due to microstrain. The crystallite sizes of the metal particles were thus extracted from the XRD data using BGMN's ‘GrainSize’ function. A flexible approach was used, where the diffraction reflexes resulting from the metal particles were fitted with one or two components, corresponding to a uniform or broader size distribution, depending on which yielded a better fit. While the metal's contribution was fitted with no texturing or anisotropy assumed, the carbon support was fitted with anisotropic peak broadening to allow it to describe the complex structure of the CB under varying laser parameters. For batch refinements of the whole dataset, an automated fitting procedure has been developed, employing a combination of BGMN and Python scripts. Each XRD pattern was fitted with a one‐component and a two‐component model. The decision for which model to choose for every pattern was based on a χ^2^‐value threshold, where a decrease of at least 10% of the χ^2^‐value was taken to justify a two‐model fit. This coincided well with the manual decisions taken to rectify the ‘W’‐shaped peak mismatch. Only in three cases human intervention was needed, namely the samples Pt‐0.8W0.43m s^−1^ and Pd‐4W0.22m s^−1^, where a two‐component fit was needed to correct the shape mismatch, and the sample Ir‐0.14W1.30m s^−1^, where no Iridium reflexes have been discernible. All Rietveld refinements systematically underestimate the intensity of the (1,1,1) reflection across all investigated metals, suggesting a preferential orientation or prevalence of this crystal plane in the laser‐induced particles. While this mismatch in intensity could have been reduced by incorporating spherical‐harmonic anisotropy corrections into the refinement models, such corrections were deliberately omitted to avoid overfitting. Additionally, their inclusion would only have led to minimal changes in the derived structural parameters, without substantially altering the overall conclusions. All data and the code used for automated Rietveld refinements are available at the following link: https://github.com/MattisGossler/XRDanalysis/tree/main/Examples/AutomaticBimodalFitting

### 4.5 HAADF‐STEM Measurements

High‐angle annular dark‐field scanning transmission electron microscopy images of the synthesized nanomaterials were obtained using a Thermo Fischer Scientific Talos F200i transmission electron microscope operated at an acceleration voltage of 200 kV. The nanomaterials were drop‐cast onto holey‐carbon TEM grids and were cleaned prior to measurements by exposing them to remote air plasma for 1 min (PIE Scientific Tergeo EM plasma cleaner). Several images were acquired at high and low magnifications for obtaining statistically relevant information about size distributions of particles. Complementary to this, qualitative elemental composition was assessed using an Energy‐dispersive X‐ray spectroscopy (STEM‐EDXS) technique with a Dual Bruker XFlash 6|100 EDS detector.

The software ImageJ was used to extract the Ferret diameter of 200 to 300 particles per sample, by approximating each particle with an ellipse and evaluating the longest distance between any two points along the boundary. From the Ferret diameter, the mean Sauter diameter was calculated:

$$D_{S}=\frac{\sum {d_{F}}^{3}}{\sum {d_{F}}^{2}}$$

which compares the volume of an ensemble of particles to its surface area and was thus especially important in catalysis.

### 4.6 SFC‐ICP‐MS Measurements

Online Pt dissolution experiments were performed using an SFC setup coupled to ICP‐MS (Nexion 350X, PerkinElmer) by Tygon tubing. The ICP‐MS performance was optimized daily, using a NexION Setup Solution (Perkin Elmer). Pt dissolution was quantified using a four‐point calibration curve (0, 1, 5, 10 µg L^−1^) prepared and measured daily, prior to the online measurements. A 10 µg L^−1^ 187Re solution was used as internal standard (Certipur ICP‐MS standard, Merck). The automated SFC setup employed in this work was described in detail elsewhere [18]. The difference was that the SFC outlet did not have an inert gas pocket around it. The electrolyte used for measurements was 0.1 м HClO4, prepared by diluting 70% HClO4 (Suprapur, Merck) with ultrapure water (18.2 MΩ·cm, Milli‐Q, Merck) directly prior to measurements. The measurement protocol consisted of 40 fast CVs (200 mV·s^−1^) and two slow CVs (10 mV·s^−1^) between 0.05 VRHE and 1.5 VRHE, separated by a potentiostatic hold at 0.05 VRHE. The hold was also applied before fast CVs and after slow CVs.

### 4.7 RDE Measurements

The oxygen reduction reaction (ORR) activity was evaluated using a rotating disk electrode (RDE) setup in a conventional three‐electrode electrochemical cell. A glassy carbon electrode (5 mm diameter) served as the working electrode, a graphite rod as the counter electrode, and an Ag/AgCl (saturated KCl) electrode as the reference. The reference electrode was calibrated against a reversible hydrogen electrode (RHE), and the measured potential difference was approximately 0.265 V, in close agreement with the value calculated using the Nernst equation.

Measurements were conducted for samples prepared at scan speeds of 0.11, 0.43, and 0.86 m s^−1^, each with a Pt loading of 10 µg cm^−^^2^. The sample prepared at 1.30 m s^−1^ was tested with a reduced Pt loading of 5 µg cm^−^^2^. Catalyst ink was prepared by dispersing the catalyst in a mixed solvent of deionized water, isopropanol, and ethanol (volume ratio 3:1:1), with a Nafion ionomer content corresponding to an I/C ratio of 0.05. The mixture was sonicated using a probe for 10 min to ensure uniform dispersion. Subsequently, 12 µL of the ink was drop‐cast onto the glassy carbon electrode and dried at room temperature.

All electrochemical measurements were performed in 0.1 м HClO_4_ at room temperature. Prior to ORR measurements, the electrolyte was first purged with high‐purity Ar for at least 20 min to record background currents. The system was then purged with high‐purity O_2_ for an additional 20 min, and cyclic voltammetry (CV) was subsequently conducted in the O_2_‐saturated electrolyte from 0.06 to 1.2 V (vs. RHE) at a scan rate of 50 mV s^−1^ under rotation speeds ranging from 400 to 1600 rpm. The measured current density was normalized to the geometric surface area of the working electrode.

### 4.8 Thermal Imaging

Infrared imaging was conducted to estimate the temperatures resulting from the laser radiation within the thin films. A thermal camera (FLIR A35, Flir Teledyne) has been used, and the data was analyzed using the software ‘Flir Research Studio’. The camera has a maximal frame rate of 60 Hz, a resolution of 320 × 256 pixels, and a temperature calibration that allows measurements of temperatures up to 704°C. It has been placed in the laser engraver approximately 10 cm from the sample. During the laser treatment, the sample was recorded, and the impact of the laser beam was captured by extracting the peak temperature at every frame within the region of interest, where the laser beam hit the sample. The temporal temperature evolution was tracked at a constant point on the irradiated sample to extract heating and cooling rates.

## Conflicts of Interest

The authors declare no conflicts of interest.

## Supporting information




**Supporting File**: smll72083‐sup‐0001‐SuppMat.docx.

## Data Availability

The data that support the findings of this study are available in the supplementary material of this article.
