# Supplementary material for: Accelerated Catalyst Development via Kinetically Controlled Solid‐State Laser Synthesis and Automated Electrochemical Screening
Source: Small. 2025 Dec 26;22(10):e11035. doi: 10.1002/smll.202511035 (PMC12910419; doi:10.1002/smll.202511035)
Supplement: Supplementary file 1 — Supporting File: smll72083‐sup‐0001‐SuppMat.docx. [file SMLL-22-e11035-s001.docx]

Supporting Information

**Accelerated Catalyst Development via Kinetically Controlled Solid-State Laser Synthesis and Automated Electrochemical Screening**

*Mattis Goßler*, Huize Wang*, Joanna Przybysz, Faisal Gabi Sameer Aldabain, A. Lucía Morales, Andreas Körner, Andreas Göpfert, Andreas Hutzler, Serhiy Cherevko, Marc Ledendecker**

Supplementary Figures


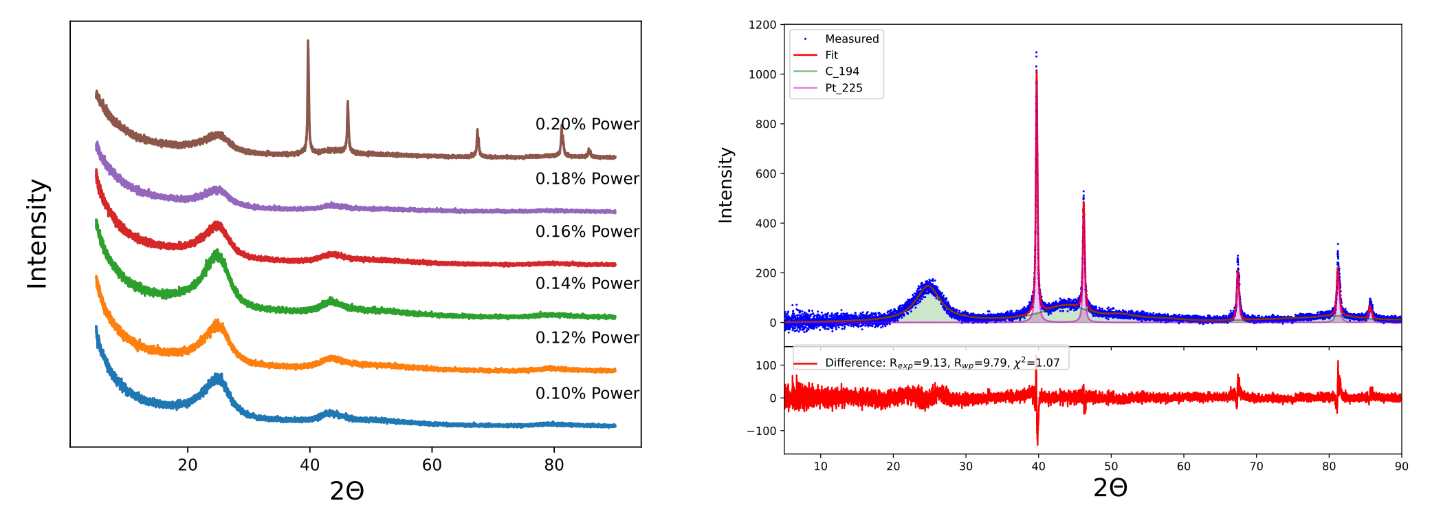


Figure S 1 Demonstration of the lack of size control observed when using K_2_PtCl_4_ as a precursor in the synthesis process. On the right: variation of power at the same level of scanning speed (100% of maximum speed) shows uncontrolled crystallization kinetics with incremental power variation (from 0.18 % to 0.20 % of maximum power). On the right: Rietveld refinement of the catalyst produced at 0.20% of maximum power; the fitted crystallite size corresponds to 37.3 nm.


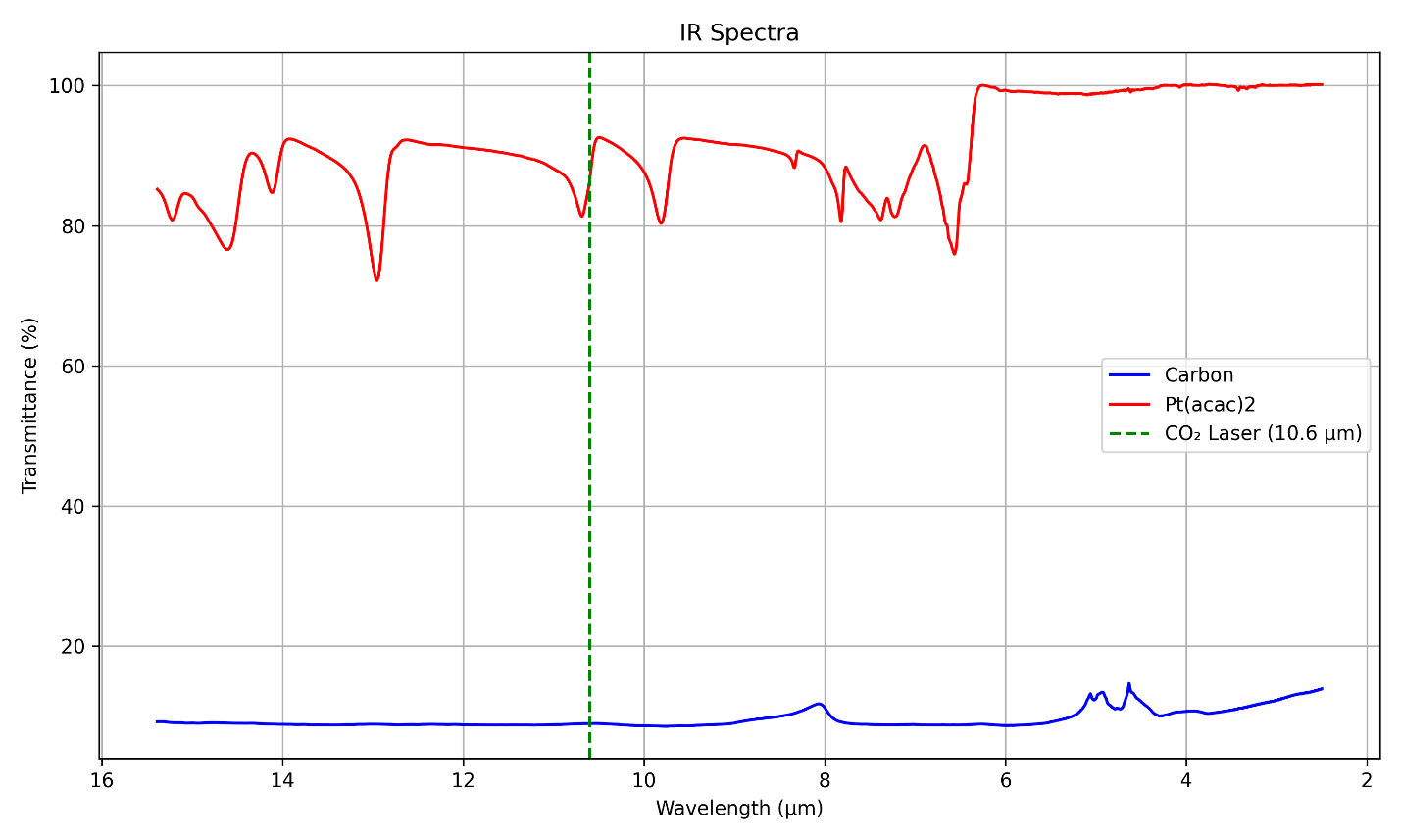


Figure S 2 Transmittance in the infrared range, showing larger absorption of the carbon support and lesser absorption of the metal precursor


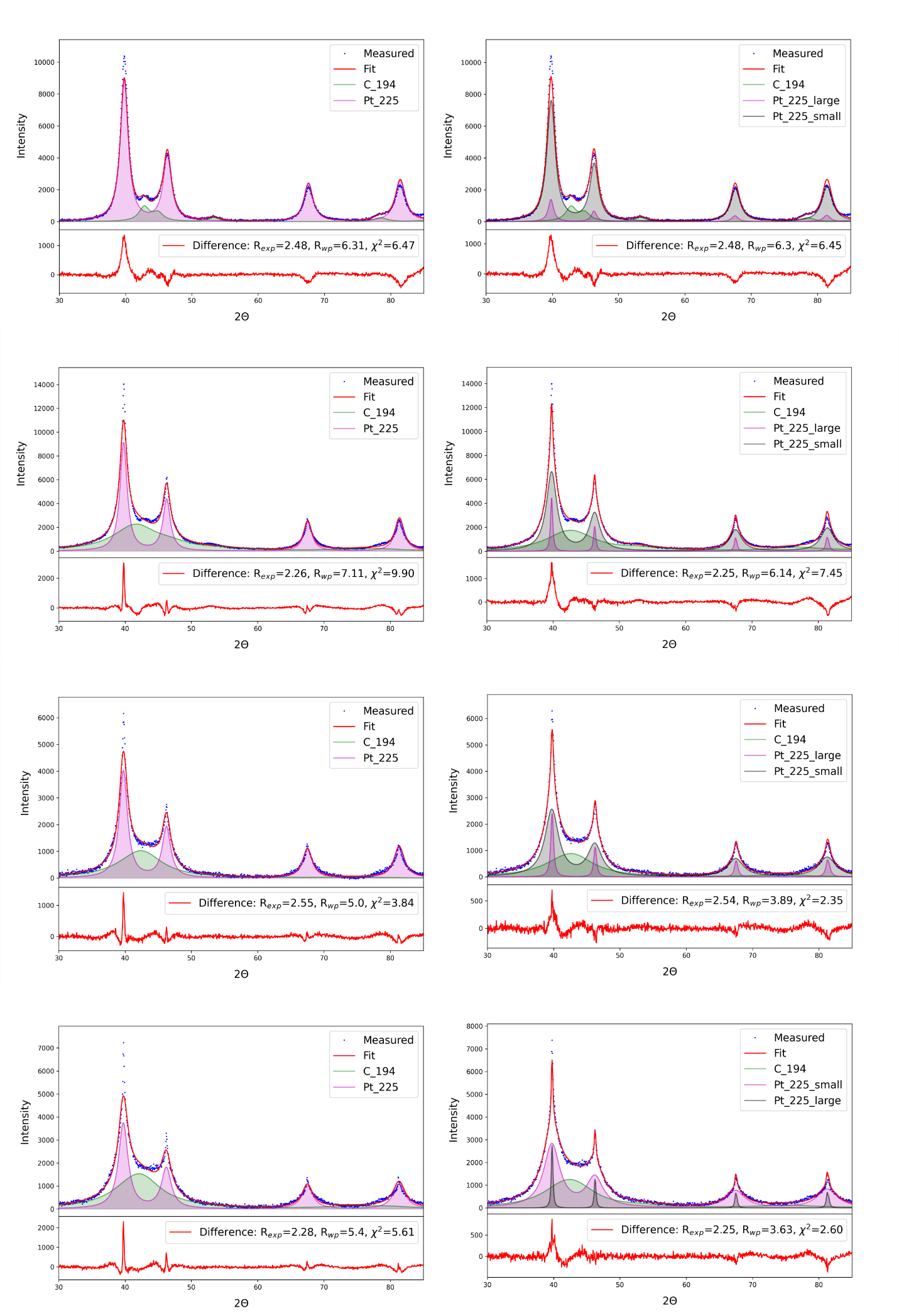


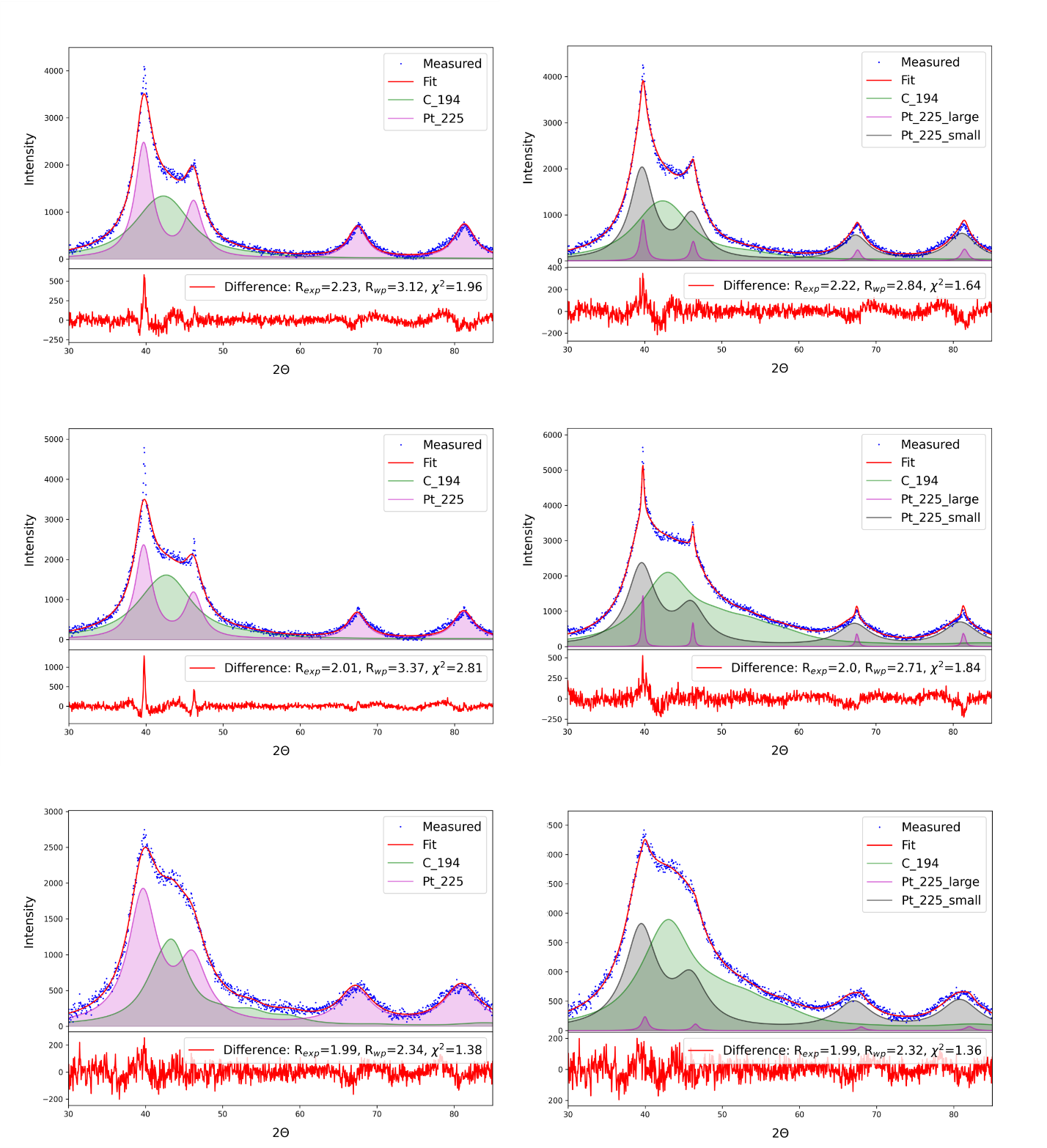


Figure S 3 Comparison of the Rietveld refinements using one (left column) or two (right column) platinum components for the velocities (from top to bottom) 0.11 m/s, 0.22 m/s, 0.43 m/s, 0.65 m/s at 0.14 W of power. Using just one component yields a mismatch of the profile of the reflex shapes for all intermediate fluences, indicated by a ‘W’ shape of the error curve. Using two components, this is mitigated, and just the intensity of the (1,1,1) peak is systematically underestimated. For very high and low fluences, the crystallite size populations are homogeneous, and a second metal component does not significantly improve the refinement.


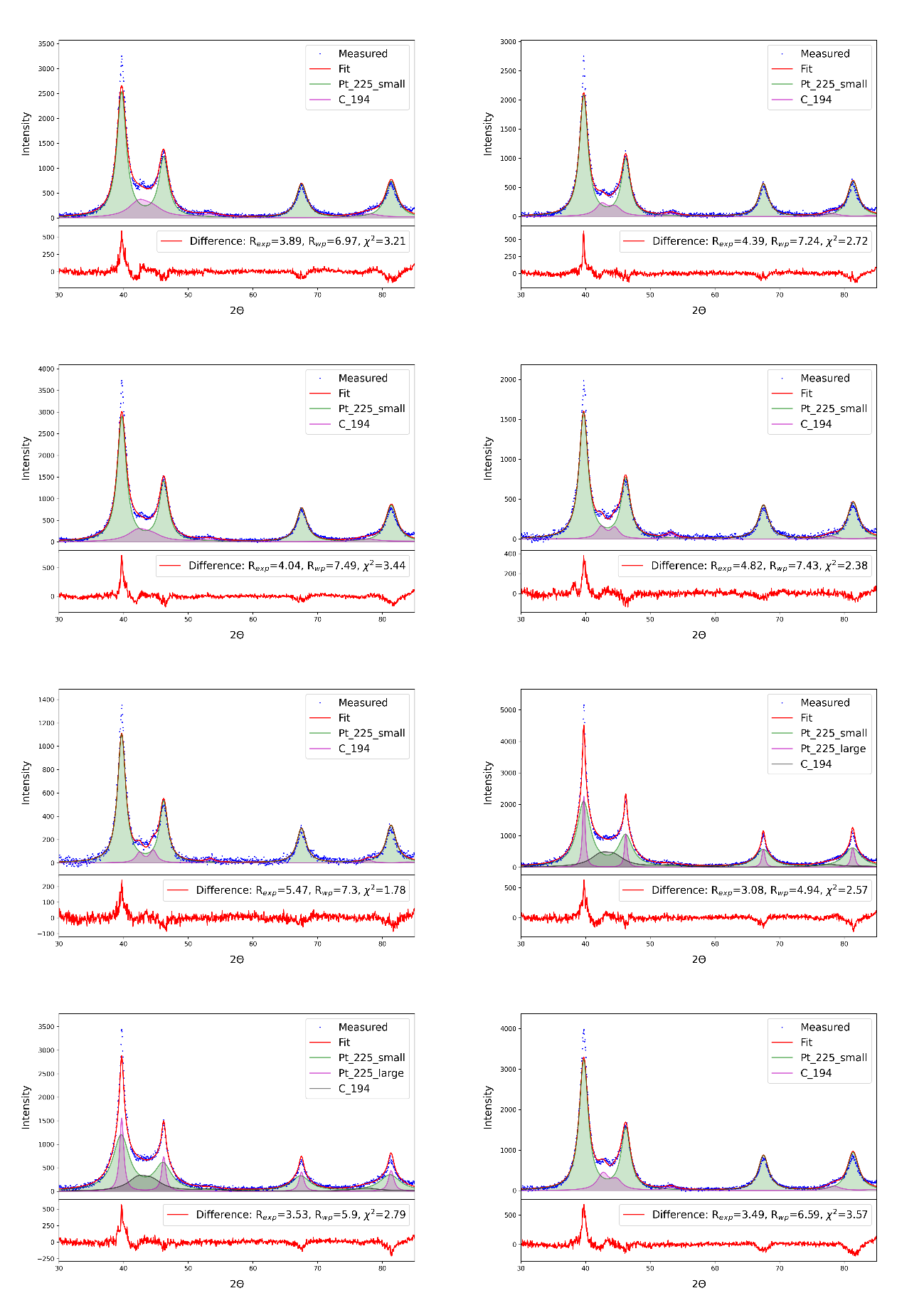


Figure S 4 Rietveld refinements of the produced platinum catalysts at the following conditions (from top left to bottom right): 8 W/1.30 m/s; 8 W/0.86 m/s; 8 W/0.43 m/s; 8 W/0.22 m/s; 8 W/0.11 m/s; 4 W/1.30 m/s; 4 W/0.86 m/s; 4 W/0.43 m/s


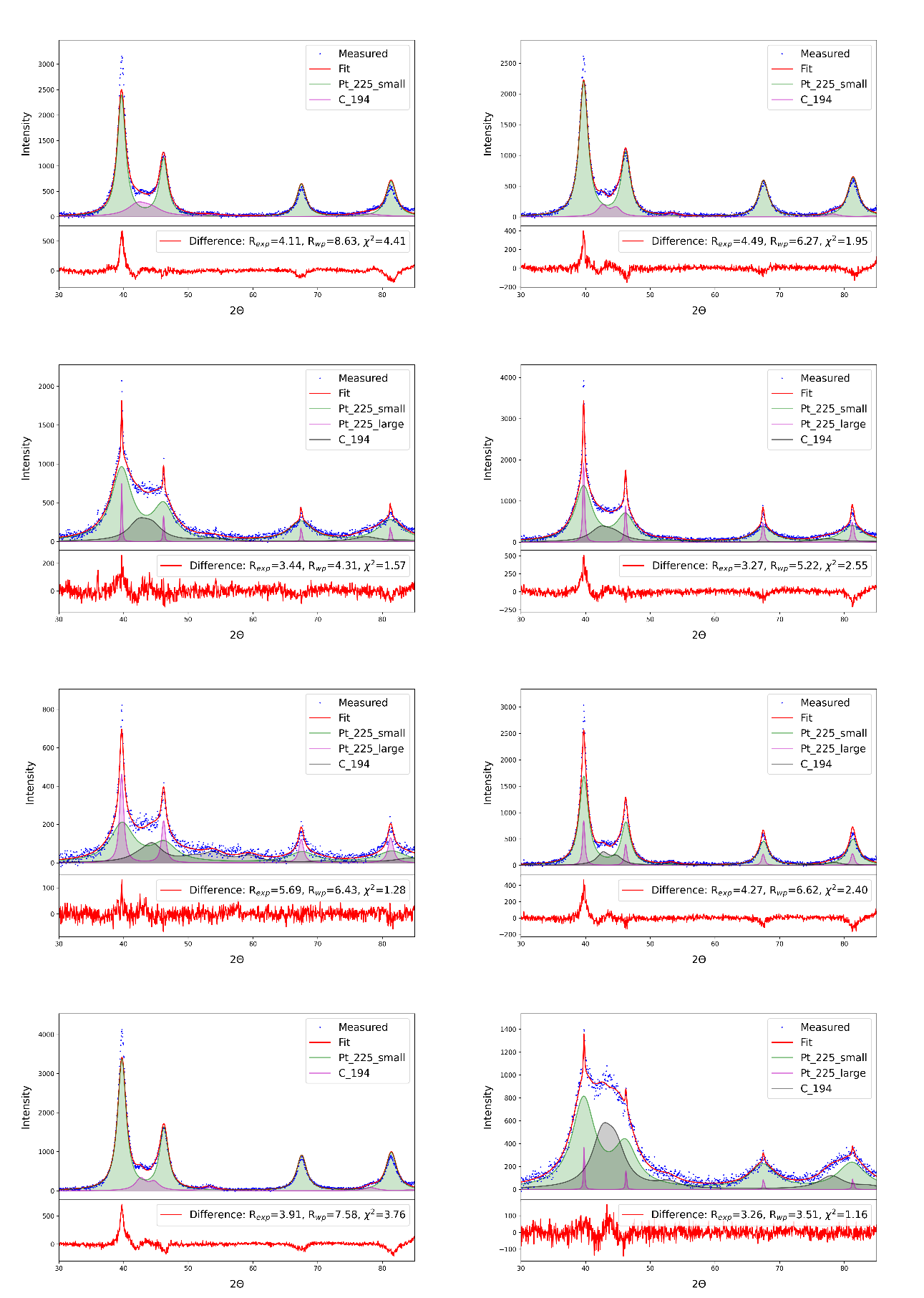


Figure S 5 Rietveld refinements of the produced platinum catalysts at the following conditions (from top left to bottom right): 4 W/0.22 m/s; 4 W/0.11 m/s; 0.8 W/1.30 m/s; 0.8 W/0.86 m/s; 0.8 W/0.43 m/s; 0.8 W/0.22 m/s; 0.8 W/0.11 m/s; 0.4 W/1.30 m/s


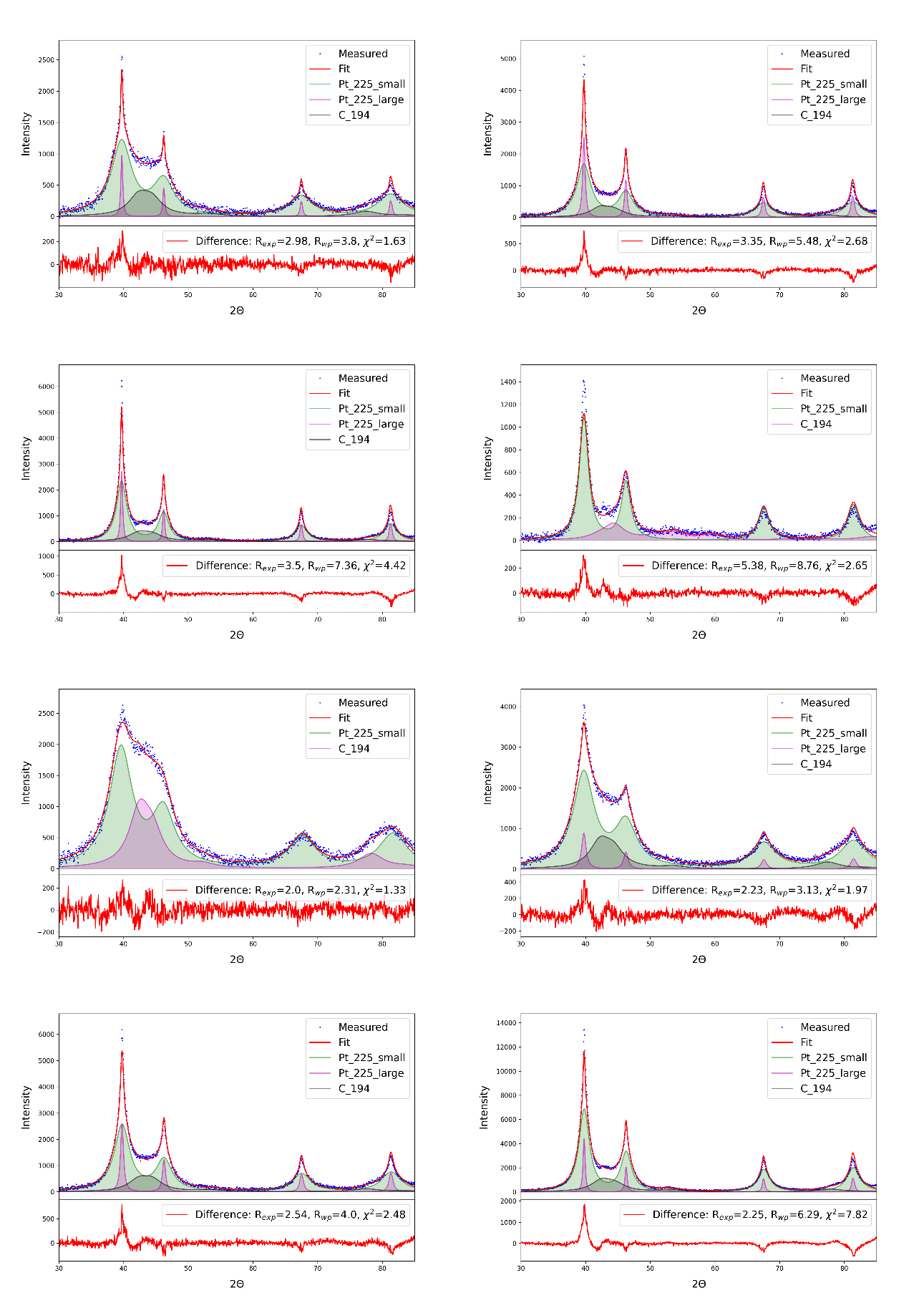


Figure S 6 Rietveld refinements of the produced platinum catalysts at the following conditions (from top left to bottom right): 0.4 W/0.86 m/s; 0.4 W/0.43 m/s; 0.4 W/0.22 m/s; 0.4W/0.11 m/s; 0.14 W/1.30 m/s; 0.14 W/0.86 m/s; 0.14 W/0.43 m/s; 0.14 W/0.22 m/s


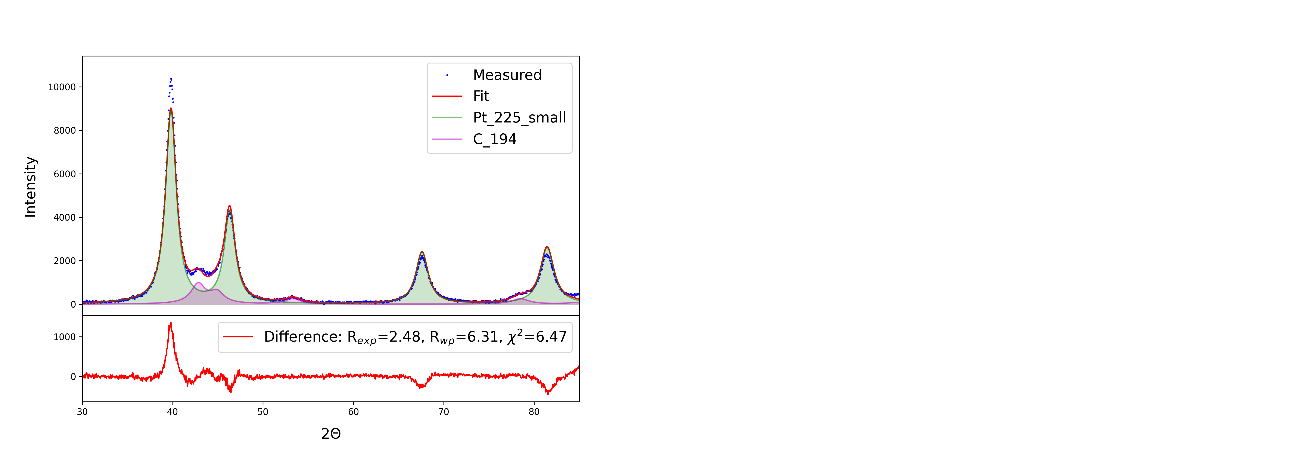


Figure S 7 Rietveld refinements of the produced platinum catalysts at the following conditions (from top left to bottom right): 0.14 W/0.11 m/s


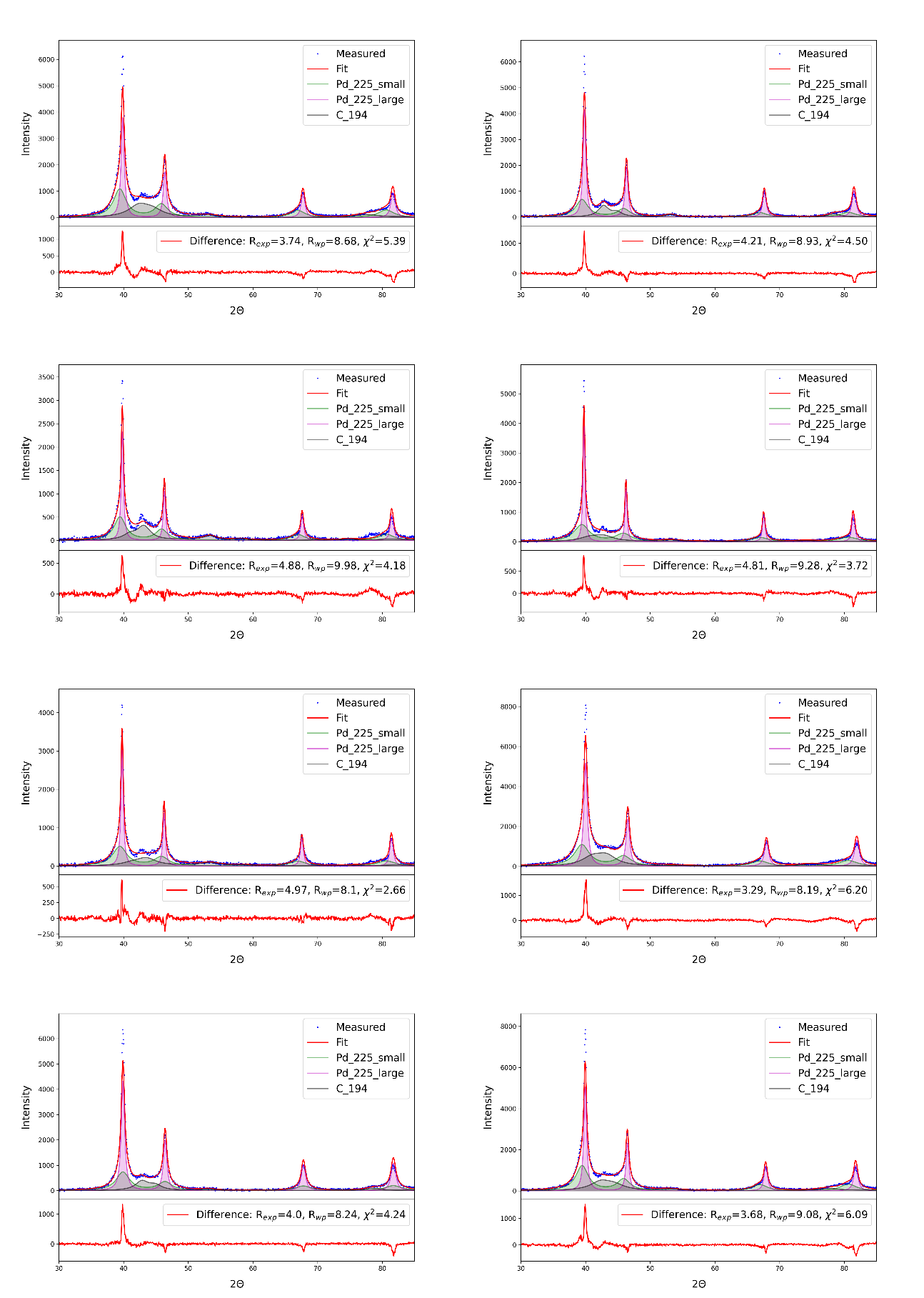


Figure S 8 Rietveld refinements of the produced palladium catalysts at the following conditions (from top left to bottom right): 8 W/1.30 m/s; 8 W/0.86 m/s; 8 W/0.43 m/s; 8 W/0.22 m/s; 8 W/0.11 m/s; 4 W/1.30 m/s; 4 W/0.86 m/s; 4 W/0.43 m/s


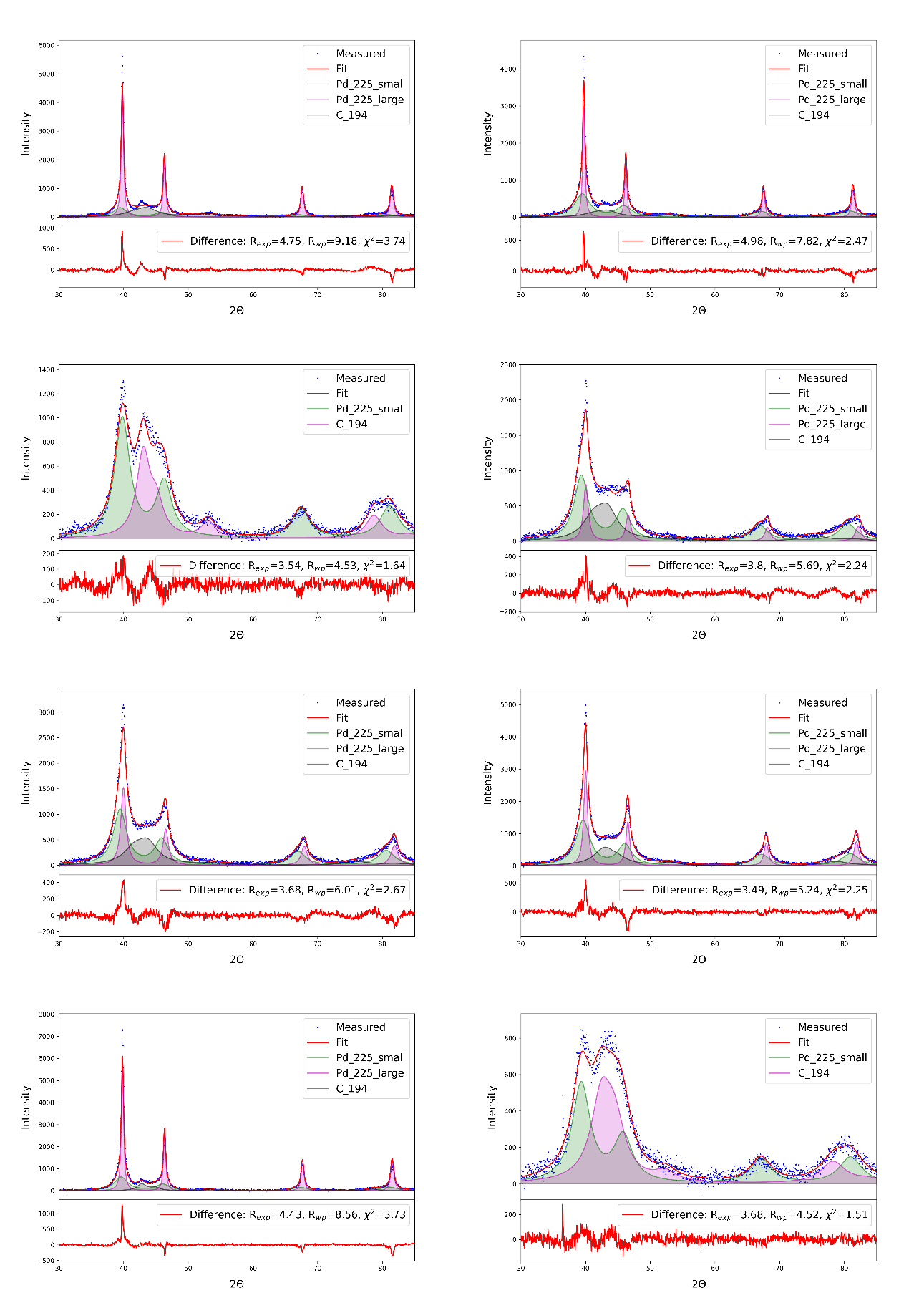


Figure S 9 Rietveld refinements of the produced palladium catalysts at the following conditions (from top left to bottom right): 4 W/0.22 m/s; 4 W/0.11 m/s; 0.8 W/1.30 m/s; 0.8 W/0.86 m/s; 0.8 W/0.43 m/s; 0.8 W/0.22 m/s; 0.8 W//0.11 m/s; 0.4 W/1.30 m/s


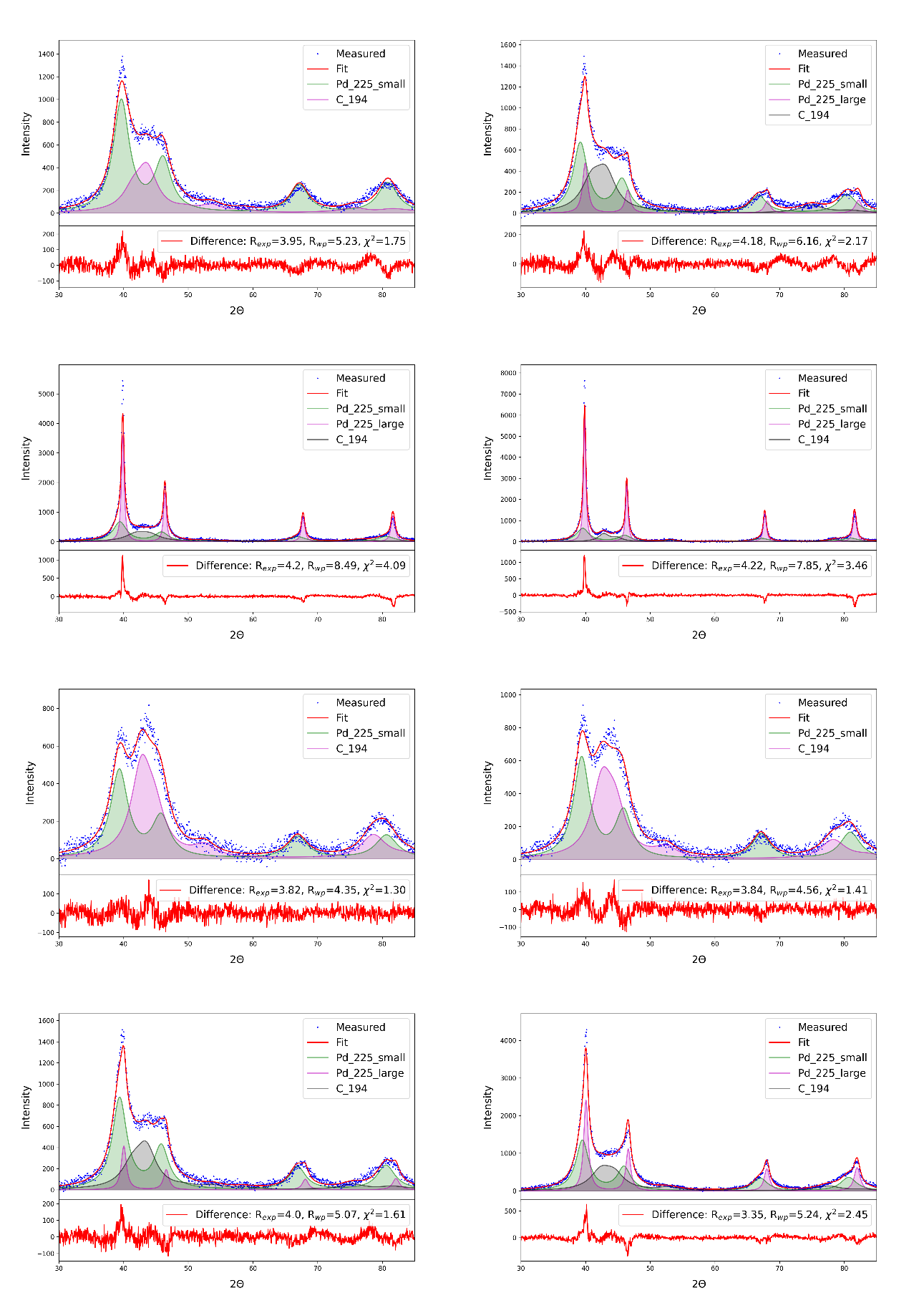


Figure S 10 Rietveld refinements of the produced palladium catalysts at the following conditions (from top left to bottom right): 0.4 W/0.86 m/s; 0.4 W/0.43 m/s; 0.4 W/0.22 m/s; 0.4W/0.11 m/s; 0.14 W/1.30 m/s; 0.14 W/0.86 m/s; 0.14 W/0.43 m/s; 0.14 W/0.22 m/s


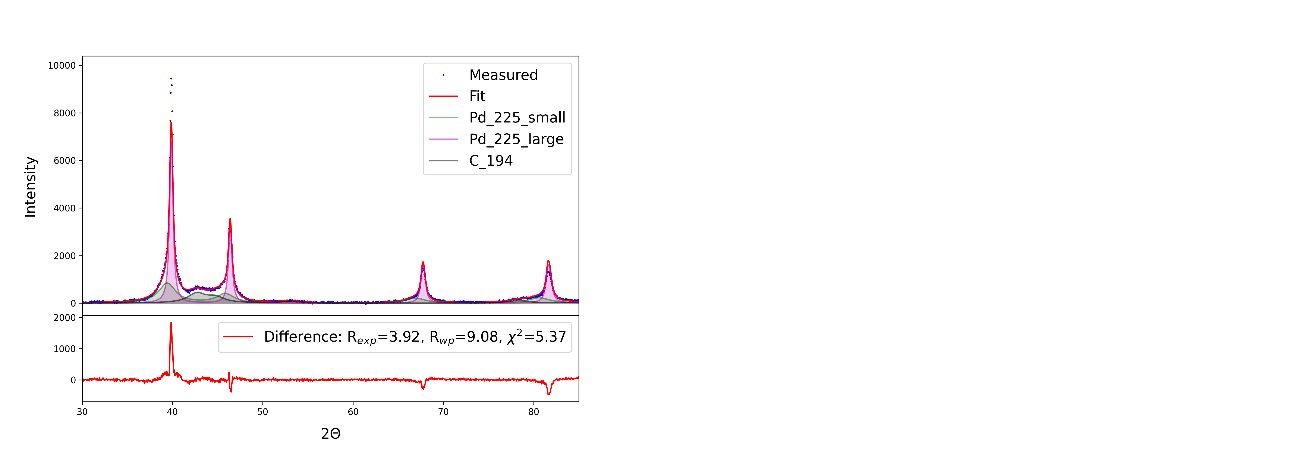


Figure S 11 Rietveld refinements of the produced palladium catalysts at the following conditions (from top left to bottom right): 0.14 W/0.11 m/s


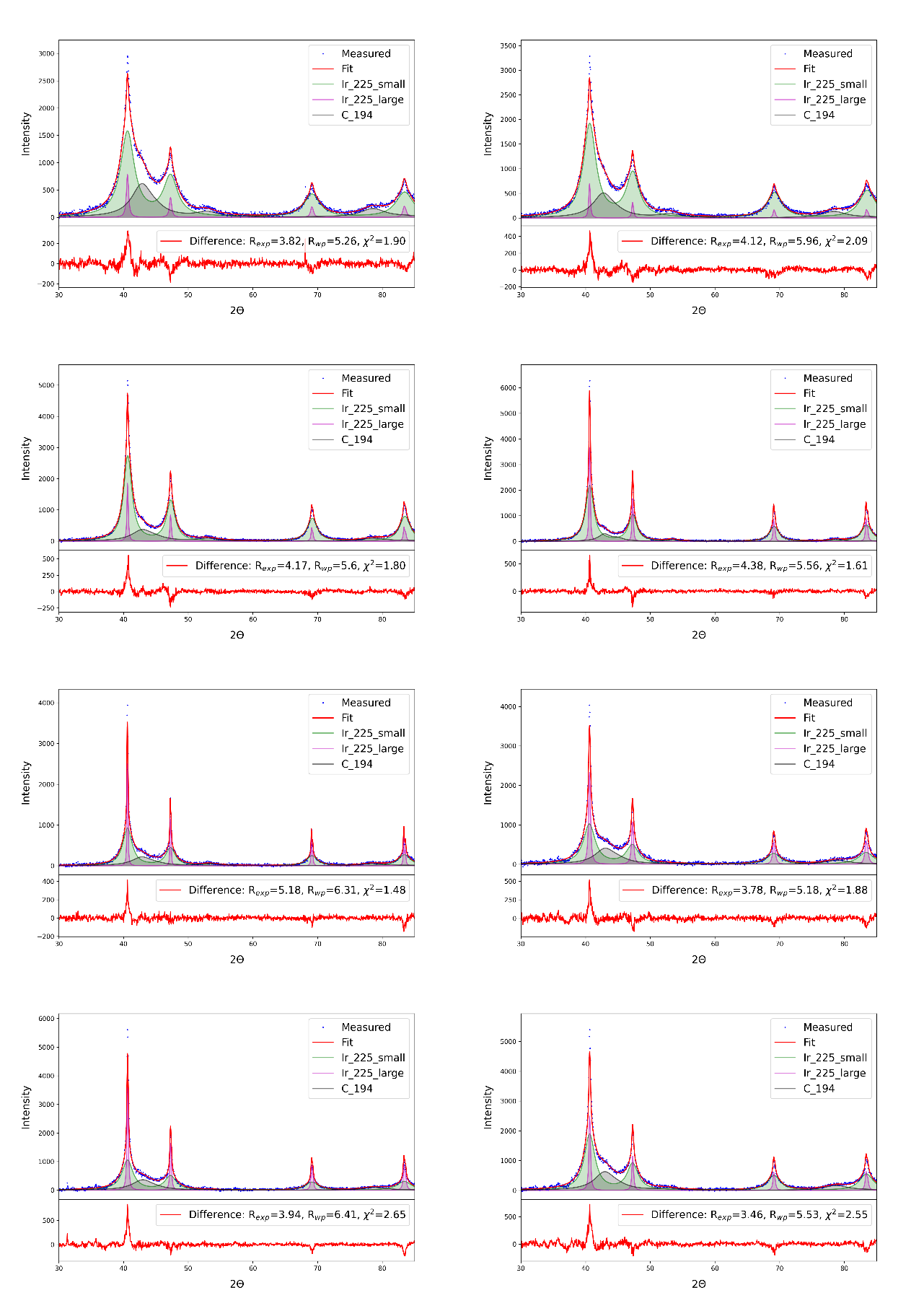


Figure S 12 Rietveld refinements of the produced iridium catalysts at the following conditions (from top left to bottom right): 8 W/1.30 m/s; 8 W/0.86 m/s; 8 W/0.43 m/s; 8 W/0.22 m/s; 8 W/0.11 m/s; 4 W/1.30 m/s; 4 W/0.86 m/s; 4 W/0.43 m/s


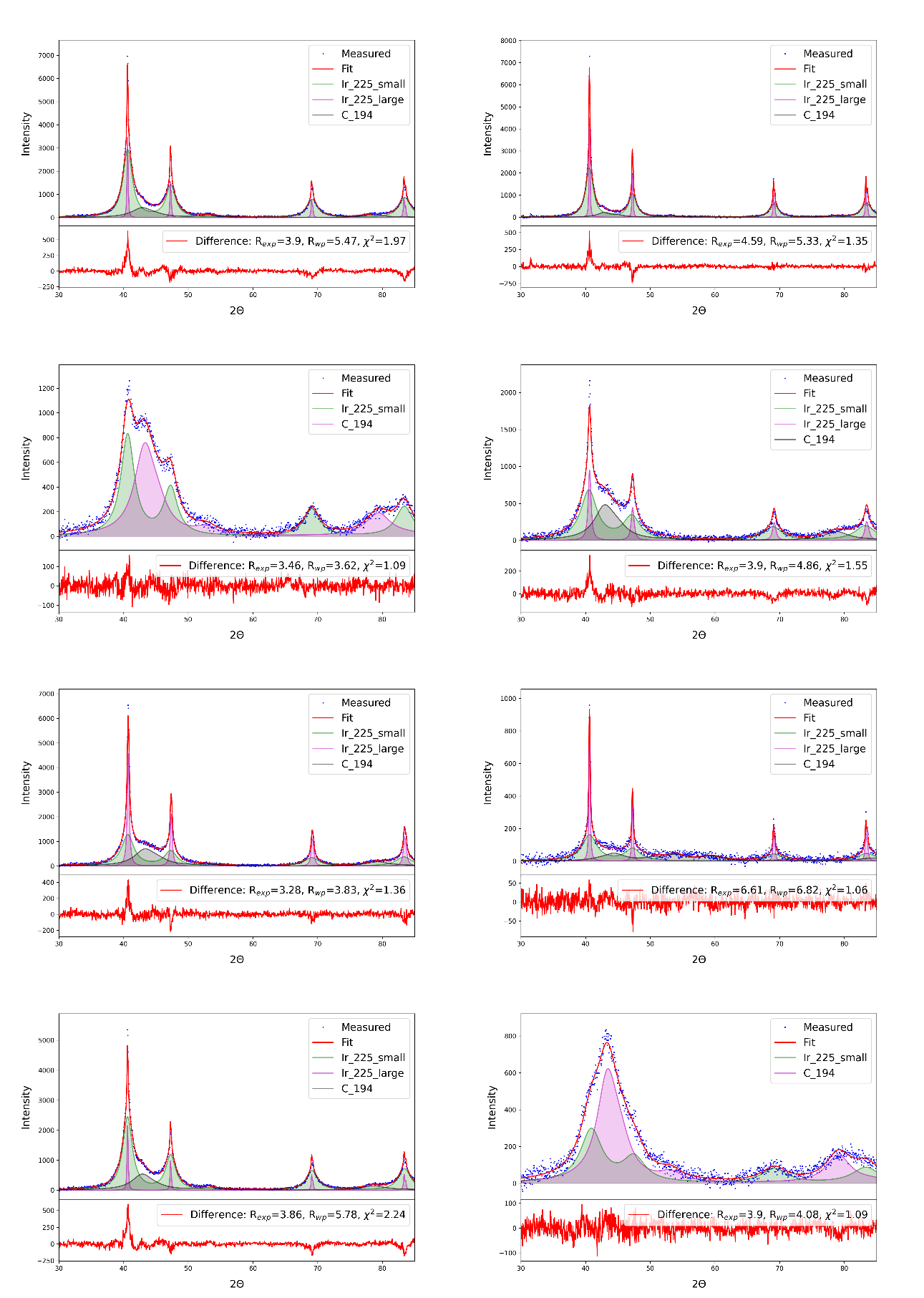


Figure S 13 Rietveld refinements of the produced iridium catalysts at the following conditions (from top left to bottom right): 4 W/0.22 m/s; 4 W/0.11 m/s; 0.8 W/1.30 m/s; 0.8 W/0.86 m/s; 0.8 W/0.43 m/s; 0.8 W/0.22 m/s; 0.8 W//0.11 m/s; 0.4 W/1.30 m/s


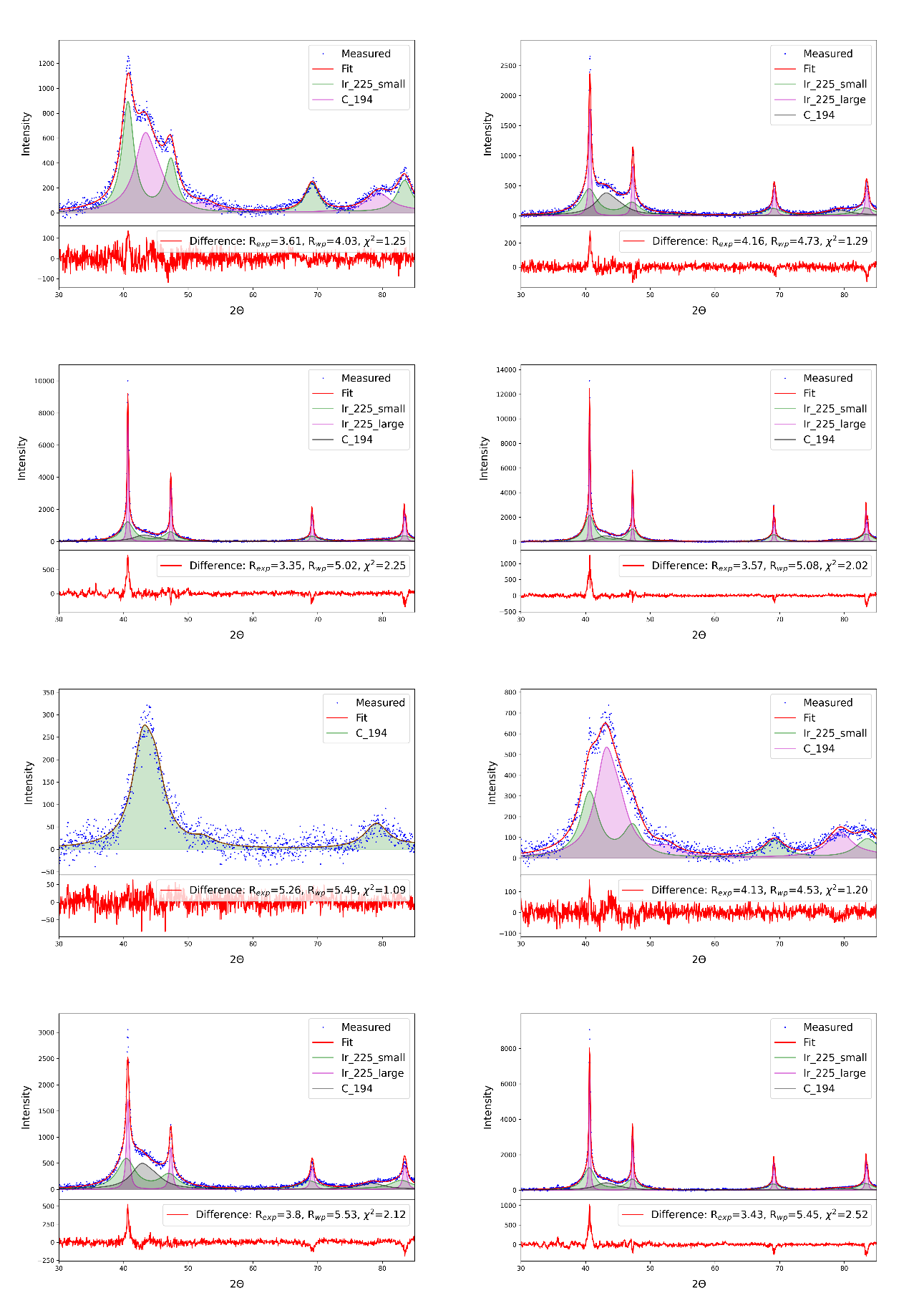


Figure S 14 Rietveld refinements of the produced iridium catalysts at the following conditions (from top left to bottom right): 0.4 W/0.86 m/s; 0.4 W/0.43 m/s; 0.4 W/0.22 m/s; 0.4W/0.11 m/s; 0.14 W/1.30 m/s; 0.14 W/0.86 m/s; 0.14 W/0.43 m/s; 0.14 W/0.22 m/s


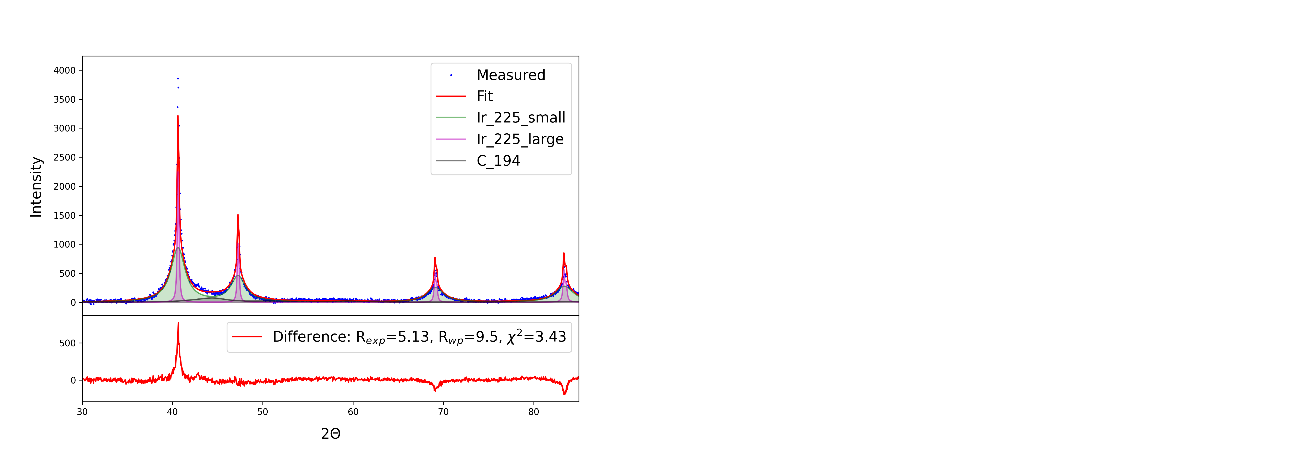


Figure S 15 Rietveld refinements of the produced iridium catalysts at the following conditions (from top left to bottom right): 0.14 W/0.11 m/s


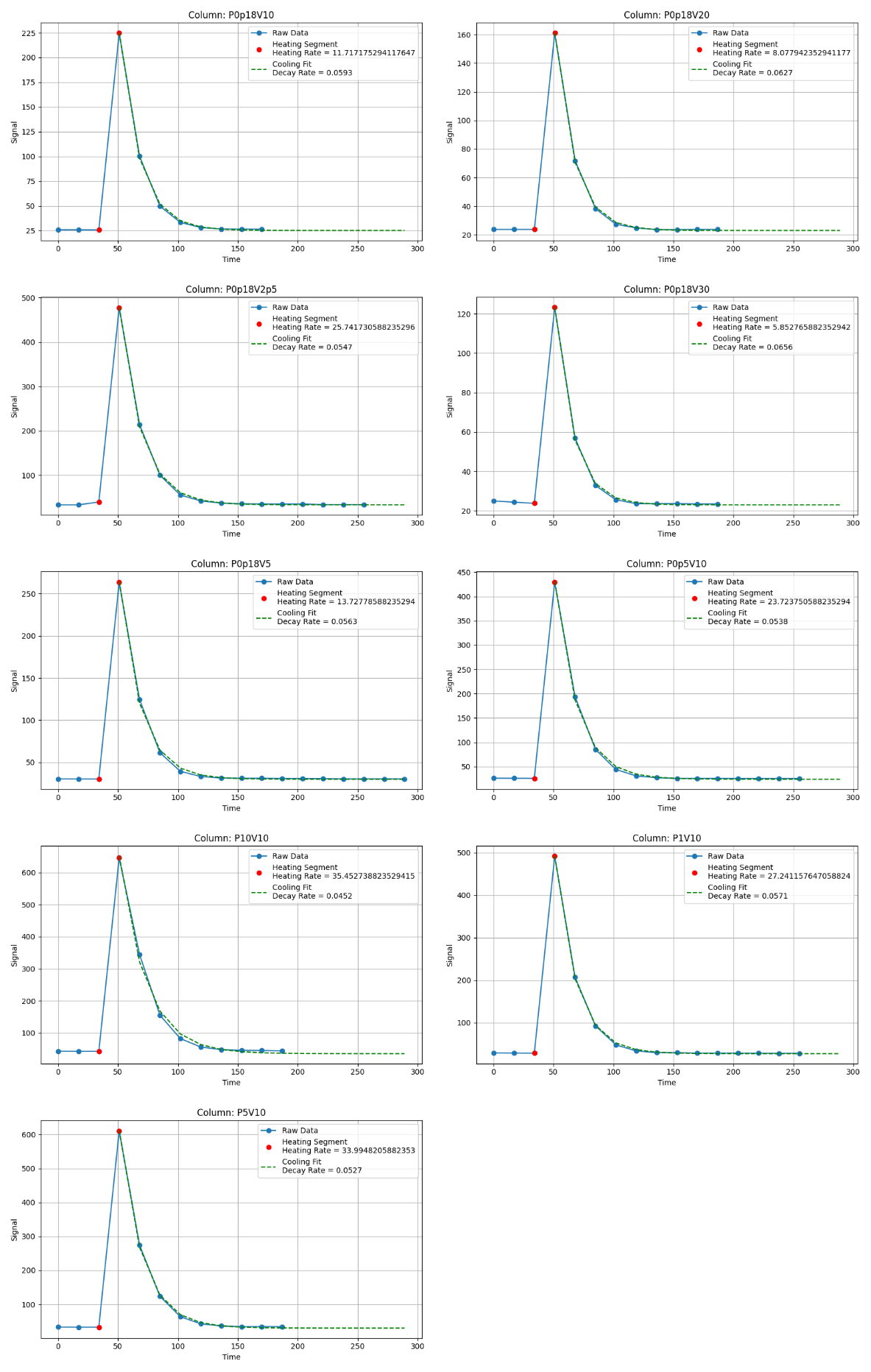


Figure S 16 Fits of heating rates and cooling halftimes, obtained from the temporal evolution of the temperature profiles, obtained from thermal imaging measurements


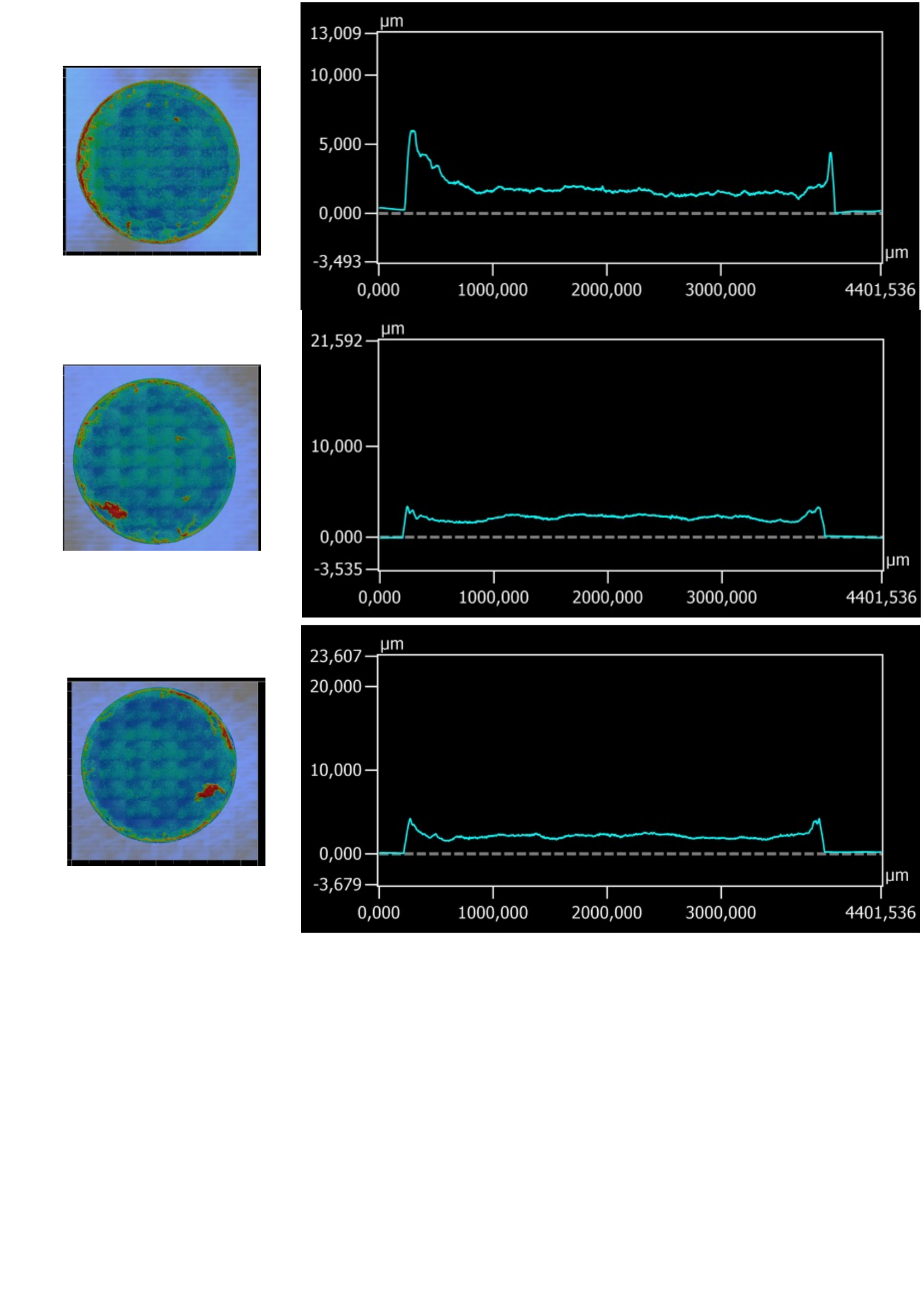


Figure S 17 Exemplary profile of 3 drop-casted catalyst spots


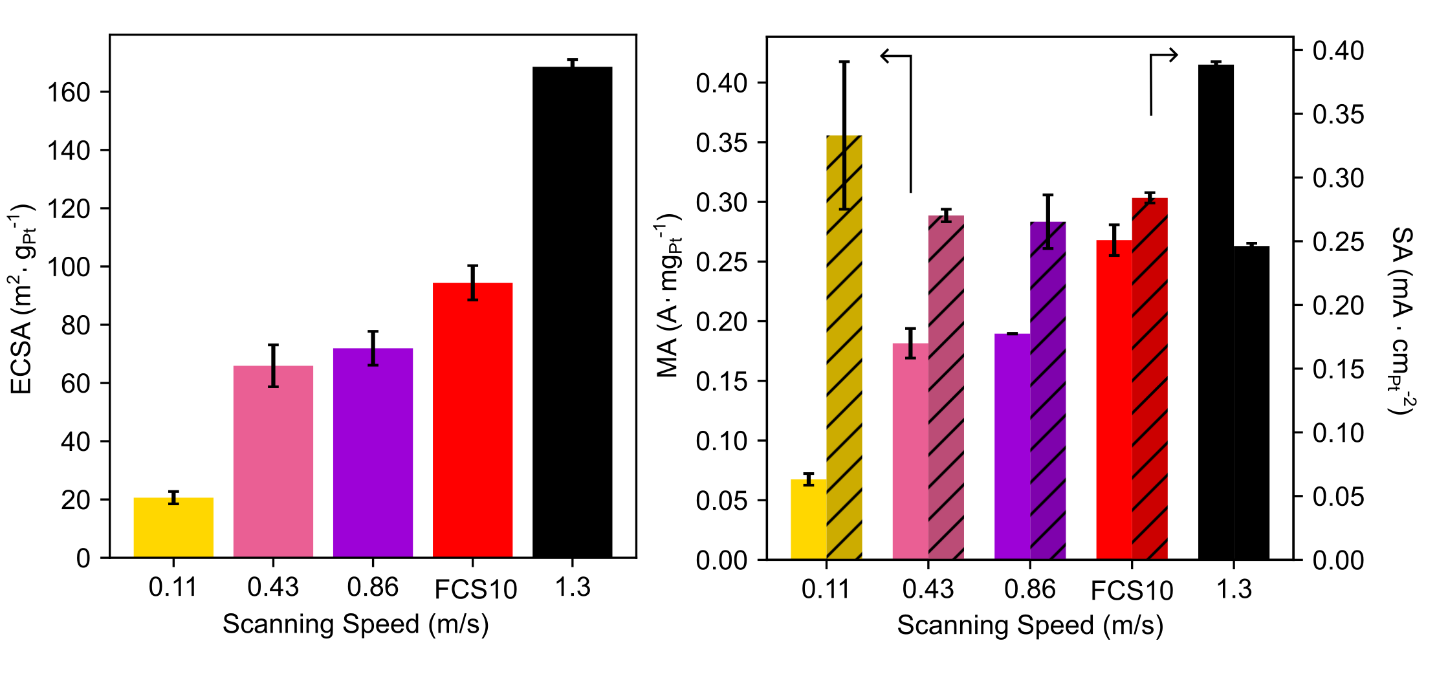


Figure S 18 Comparison of ECSA, MA, and SA, extracted from ORR polarization curves, appended with data of a commercial Pt/C catalyst (10wt% platinum on Vulcan XC 72, Fuel Cell Store), measured under the same conditions and designated as FCS10 in the Figure. The activities, as well as the measured ECSA of the benchmark, align well with the trend formed by the laser-synthesized samples.


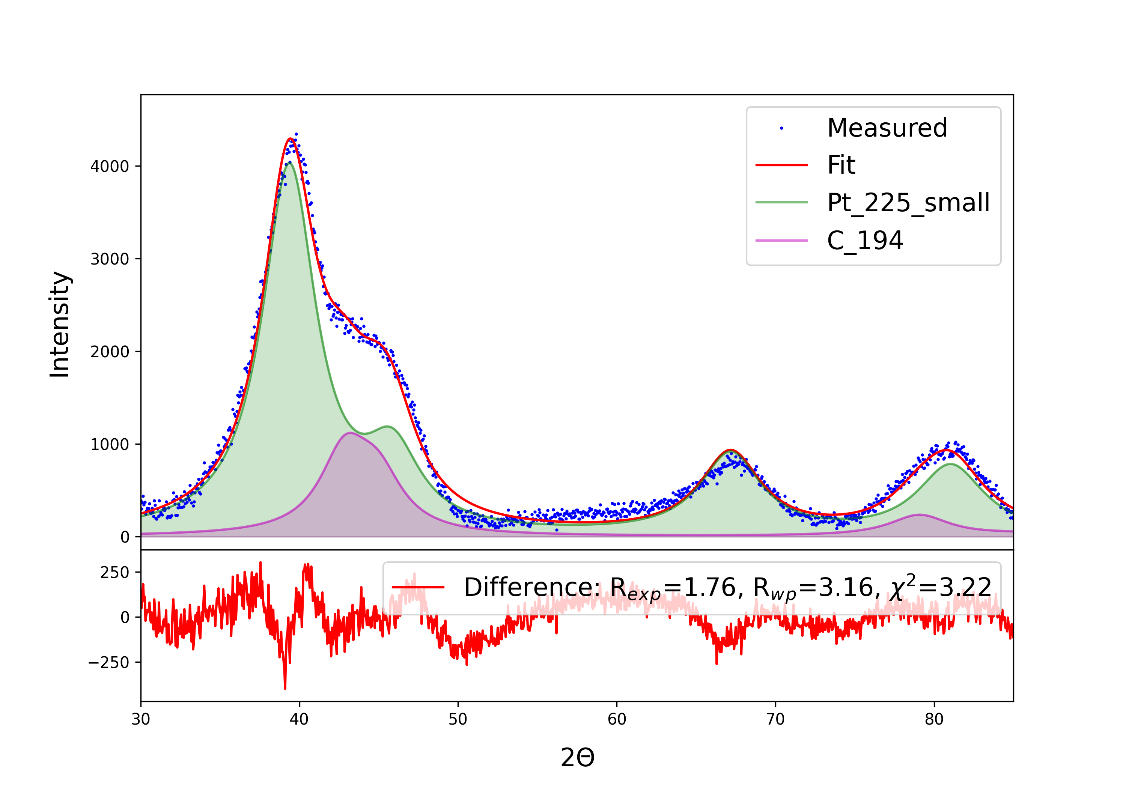


Figure S 19 XRD pattern of the commercial Pt/C catalyst FCS10 (10wt% platinum on Vulcan XC72, Fuel Cell Store), where Rietveld refinement yielded a Particle size of 1.9 nm.

Supplementary Tables

Table S 1 Crystallite sizes obtained from XRD analysis and particle sizes obtained from TEM analysis.

| Laser parameters | XRD crystallite size (nm) | TEM particle size (nm) | Deviation (%) |
| --- | --- | --- | --- |
| 0.14 W, 1.30 m/s | 1.6 | 2.1 | 24 |
| 0.14 W, 1.08 m/s | 1.8 | 2.1 | 14 |
| 0.14 W, 0.86 m/s | 2.2 | 2.6 | 15 |
| 0.14 W, 0.65 m/s | 2.7 | 3.6 | 25 |
| 0.14 W, 0.43 m/s | 3.2 | 3.9 | 18 |
| 0.14 W, 0.22 m/s | 4.2 | 5.5 | 24 |
| 0.14 W, 0.11 m/s | 5.6 | 6.0 | 7 |

Table S 2 Mean, minimal, and maximal peak temperatures extracted from thermal imaging measurements.

| Laser parameters | Mean temperature (°C) | Min. temperature (°C) | Max. temperature (°C) |
| --- | --- | --- | --- |
| 0.14 W, 1.30 m/s | 129 | 82 | 213 |
| 0.14 W, 0.86 m/s | 164 | 108 | 251 |
| 0.14 W, 0.43 m/s | 221 | 111 | 389 |
| 0.14 W, 0.22 m/s | 268 | 110 | 450 |
| 0.14 W, 0.11 m/s | 481 | 117 | 704 |
| 0.4 W, 0.43 m/s | 415 | 62 | 704 |
| 0.8 W, 0.43 m/s | 511 | 127 | 704 |
| 4 W, 0.43 m/s | 601 | 209 | 704 |
| 8 W, 0.43 m/s | 665 | 349 | 704 |

Table S 3 Heating rates and cooling halftimes, extracted from the temporal temperature evolution, are recorded at a pixel in the middle of a catalyst spot.

| Laser parameters | Heating rate  (°C/ms) | Cooling halftime  (ms) | Max. temperature (°C) |
| --- | --- | --- | --- |
| 0.14 W, 1.30 m/s | 5.852 | 10.6 | 123 |
| 0.14 W, 0.86 m/s | 8.078 | 11.1 | 161 |
| 0.14 W, 0.43 m/s | 11.717 | 11.7 | 225 |
| 0.14 W, 0.22 m/s | 13.727 | 12.3 | 263 |
| 0.14 W, 0.11 m/s | 25.742 | 12.7 | 477 |
| 0.4 W, 0.43 m/s | 23.724 | 12.9 | 430 |
| 0.8 W, 0.43 m/s | 27.241 | 12.1 | 491 |
| 4 W, 0.43 m/s | 33.995 | 13.2 | 611 |
| 8 W, 0.43 m/s | 35.453 | 15.3 | 646 |

Table S 4 Metal loadings of catalysts produced at different laser parameters, measured by ICP-OES.

| Laser parameters | Platinum mass loading (%) |
| --- | --- |
| 0.14 W, 1.30 m/s | 2.14 |
| 0.14 W, 1.08 m/s | 3.76 |
| 0.14 W, 0.86 m/s | 5.72 |
| 0.14 W, 0.65 m/s | 5.52 |
| 0.14 W, 0.43 m/s | 8.32 |
| 0.14 W, 0.22 m/s | 7.17 |
| 0.14 W, 0.11 m/s | 7.34 |

Table S 5 Lineal and Areal Fluence values, resulting from the utilized laser power and speed values.

| Laser Power (W) | Laser Velocity (m/s) | Lineal Fluence (J/m) | Areal Fluence (J/m^2^) |
| --- | --- | --- | --- |
| 0.14 | 1.30 | 0.11 | 925.93 |
| 0.4 | 1.30 | 0.31 | 2572.02 |
| 0.8 | 1.30 | 0.62 | 5144.03 |
| 4 | 1.30 | 3.09 | 25720.16 |
| 8 | 1.30 | 6.17 | 51440.33 |
| 0.14 | 0.86 | 0.17 | 1388.89 |
| 0.4 | 0.86 | 0.46 | 3858.02 |
| 0.8 | 0.86 | 0.93 | 7716.05 |
| 4 | 0.86 | 4.63 | 38580.25 |
| 8 | 0.86 | 9.26 | 77160.49 |
| 0.14 | 0.43 | 0.33 | 2777.78 |
| 0.4 | 0.43 | 0.93 | 7716.05 |
| 0.8 | 0.43 | 1.85 | 15432.10 |
| 4 | 0.43 | 9.26 | 77160.49 |
| 8 | 0.43 | 18.52 | 154320.99 |
| 0.14 | 0.22 | 0.67 | 5555.56 |
| 0.4 | 0.22 | 1.85 | 15432.10 |
| 0.8 | 0.22 | 3.70 | 30864.20 |
| 4 | 0.22 | 18.52 | 154320.99 |
| 8 | 0.22 | 37.04 | 308641.98 |
| 0.14 | 0.11 | 1.33 | 11111.11 |
| 0.4 | 0.11 | 3.70 | 30864.20 |
| 0.8 | 0.11 | 7.41 | 61728.40 |
| 4 | 0.11 | 37.04 | 308641.98 |
| 8 | 0.11 | 74.07 | 617283.95 |
